# Supplementary material for: The Cell-Surface Marker Sushi Containing Domain 2 Facilitates Establishment of Human Naive Pluripotent Stem Cells
Source: Stem Cell Reports. 2019 Apr 25;12(6):1212–22. doi: 10.1016/j.stemcr.2019.03.014 (PMC6565611; doi:10.1016/j.stemcr.2019.03.014)
Supplement: Document S1. Supplemental Experimental Procedures, Figures S1–S4, and Table S1 [file mmc1.pdf]

Stem Cell Reports, Volume *12*

Supplemental Information

The Cell-Surface Marker Sushi Containing Domain 2 Facilitates Establishment of Human Naive Pluripotent Stem Cells

Nicholas Bredenkamp, Giuliano Giuseppe Stirparo, Jennifer Nichols, Austin Smith, and Ge Guo

INVENTORY OF SUPPLEMENTAL INFORMATION

Supplemental methods

Cell culture procedures, reverse transcription and RT-qPCR, immunofluorescence staining, and flow cytometry are described.

Table S1 RT-qPCR assays

Supplemental figure legends

Figure S1 Cell surface marker expression by RNA-seq analysis, Relates to Figure 1

Figure S2 Cell surface marker expression in human embryos and naïve hPSC, Relates to Figure 1

Figure S3 Capacitation of human naïve PSC, Relates to Figure 1

Figure S4 Application of SUSD2 for identification and purification of human naïve PSC during resetting and reprogramming, Relates to Figure 2 and Figure 4

Supplemental references

SUPPLEMENTAL METHODS

Human naïve PSC culture

Unless otherwise indicated, human naïve PSC were propagated in PXGL medium on irradiated MEF feeders. ROCK inhibitor (Y-27632) was added during replating. Geltrex (10 μg per cm^2 surface area) was added to media during plating for feeder-free expansion.

PXGL medium: N2B27 (in house) supplemented with 1 μM PD0325901 (P), 2μM XAV939 (X), 2 μM Gö6983 (G) and 10 ng/ml human LIF (L).

t2iLGö medium: N2B27 supplemented with 1 μM PD0325901, 1 μM CHIR99021 (2i), 2 μM Gö6983 (Gö) and 10 ng/ml human LIF (L).

N2B27 medium (1L): 487 ml DMEM/F12 (Sigma, D6421), 487 ml Neurobasal (Thermo Fisher, 21103049), 10 ml B27 (Thermo Fisher, 17504044), 5 ml N2 (made in house), 10 ml L-Glutamine (200mM, Thermo Fisher, 25030024), 1ml 0.1M β-mercaptoethanol (Sigma, M3148)

N2 (made in house): DMEM/F12 basal medium supplemented with 0.4 mg/ml Insulin (Sigma, 19278), 10 mg/ml Apo-transferrin (eBioscience, ABC2553), 3 μM Sodium selenite (Sigma, S5261), 1.6 mg/ml Putrescine (Sigma, P5780) and 2 μg/ml Progesterone (Sigma, P8783).

Chemical Resetting

Conventional human PSC were seeded at $1\times10^4/cm^2$ onto irradiated MEF feeders in E8 medium. Two days later (day 0) medium was changed to PDL/HDACi (N2B27 with 1μM PD0325901, 10ng/ml human LIF and 1mM valproic acid sodium salt (VPA, Sigma, P4543) (HDACi)). Following 3 days in PDL/HDACi, medium was changed to

PXGL and refreshed daily for a further 10-11 days before passaging to establish naïve PSC cultures in PXGL medium on irradiated MEF feeders.

Somatic cell reprogramming

Human dermal fibroblast (HDF) cells were seeded onto gelatin coated plates in fibroblast culture medium at a density of 1×10^4 cells per cm^2. The next day Sendai virus vectors (Fusaki et al., 2009) were applied for 24 hours and the culture was refreshed with fibroblast culture medium. Following 3 or 4 more days of culture in fibroblast culture medium, cells were passaged onto irradiated mouse MEF feeders and cultured in Essential 6 (E6) supplemented with 10ng/ml FGF2 (prepared in-house) for 4 days. Cells were then switched into naïve media (PXGL+Y) to capture naïve iPSCs. Episomal reprogramming was performed with similar protocol except that reprogramming factors (*pCXLE-OCT4-shRNA(p53*), *pCXLE--SOX2-KLF4* and *pCXLE-L-MYC-LIN28* (Okita et al., 2011) were transfected into HDFs by electroporation.

Reverse transcription and RT-qPCR

Total RNA was extracted using a RNeasy Kit (Qiagen) and cDNA synthesized with SuperScript III reverse transcriptase (Thermo Fisher Scientific, 18080085) and oligo(dT) adapter primers. TaqMan assays and Universal Probe Library (UPL) probes (Roche Molecular Systems) were used to perform gene quantification.

Immunostaining

Cells were fixed with 4% PFA for 15 min at room temperature and blocked/permeabilised with 0.1% Triton X-100 and 5% donkey serum in PBS for 30

min. Incubation with primary antibodies KLF17 (Atlas Antibodies HPA024629), NANOG (R&D Systems AF1997), TFCP2L1 (R&D Systems AF5726), SUSD2 clone W5C5 (BioLegend 327401), GATA4 (Santa Cruz sc-1237), MECP2 (Cell Signalling Technology 3456T) was overnight at 4°C and secondary antibodies were added for 1 h at room temperature. For live cell staining of human cells incubation with conjugated SUSD2 clone W5C5 (SUSD2-PE, BioLegend 327406; SUSD2-FITC, Miltenyi Biotech 130-106-401), CD7-PE clone 6B7 (BioLegend 343105), CD75-FITC clone LN1 (BD Biosciences 555654), CD77-FITC clone 5B5 (BioLegend 357103), CD130-PE clone 2E1B02 (BioLegend 362003) in culture media was for 30 min before washing and imaging.

Flow cytometry

Flow cytometry analysis was carried out on a Fortessa instrument (BD Biosciences). Cell sorting was performed using a MoFlo high-speed instrument (Beckman Coulter). The following antibodies were used for flow cytometry: SUSD2 clone W5C5 (SUSD2-PE, BioLegend 327406; SUSD2-APC, BioLegend 327408) CD24-FITC (BioLegend 311104) or CD24-APC or CD24-PECy7 (eBioscience 17-0247-42 or 25-0247-41), SSEA4-APC (BioLegend 330418), CD7-APC (BioLegend 343108), CD75-eFluor 660 (eBioscience 50-0759-42), CD77-Alexa 647 (BD Biosciences 563632), CD130-PE (BioLegend 362003).

Table S1. RT-qPCR assays

GENE	ASSAY METHOD	DETAILS
ACTB	TaqMan	Assay ID: Hs01060665_g1
NANOG	TaqMan	Assay ID: Hs02387400_g1
OCT4	TaqMan	Assay ID: Hs01654807_s1
KLF4	TaqMan	Assay ID: Hs00358836_m1
KLF17	TaqMan	Assay ID: Hs00703004_s1
TFCP2L1	TaqMan	Assay ID: Hs00232708_m1
DPPA3	TaqMan	Assay ID: Hs01931905_g1
DPPA5	TaqMan	Assay ID: Hs00988349_g1
SUSD2	UPL	Forward primer: agagctggatggacctgaaa<br>Reverse primer: atgccagcatgatggagac<br>UPL probe number: 83

SUPPLEMENTAL FIGURE LEGENDS

Figure S1 Cell surface marker expression by RNA-seq analysis, Relates to Figure 1

(A) Published human naïve cell surface marker (Collier et al., 2016; O'Brien et al., 2016) transcript levels in human embryos at different stages and lineages shown, and in various naïve (pale pink bars) and conventional (green bars) hPSC extracted from an integrated single cell RNA-sequence dataset (Stirparo et al., 2018). cMOR, compacted morula; eICM, early inner cells mass; TE, trophectoderm; Epi, epiblast; PrE, primitive endoderm. (B) Epiblast to primitive endoderm average FPKM ratio for published cell surface markers and SUSD2.

Figure S2 Cell surface marker expression in human embryos and naïve hPSC, Relates to Figure 1

(A) Immunofluorescence staining for SUSD2, KLF17 and GATA4 in three E7 human blastocysts; KLF17 marks the ICM. Scale bar, 50 µm. (B) RT-qPCR analysis of general

and naïve pluripotency markers in HNES1 cells in t2iLGö or PXGL medium. (C) Top panel, flow cytometry analysis showing cell surface marker expression in conventional (SHEF6, S6), naïve chemically reset hPSC (cR-S6) and naïve embryo derived hPSC (HNES1). Bottom panel, images show bright field and immunofluorescence staining using conjugated antibodies as shown, on live naïve chemically reset hPSC (cR-H9) and naïve embryo derived hPSC (HNES1). Exposure time: CD7-PE, CD75-FITC, CD77-FITC, CD130-PE, 15s; SUSD2-FITC, 12s; SUSD2-PE, 3s. Scale bar, 100μm.

Figure S3 Capacitation of human naïve PSC, Relates to Figure 1

(A) Bright field images show colony morphology changes during capacitation of cR-S6 and HNES1 cells in N2B27-XAV medium. Scale bars, 50μm. (B) Alkaline phosphatase (AP) staining of cR-S6 and HNES1 cells following 10 days capacitation and replating and culture in PXGL for 7 days. Images show AP staining of a single well of a 12-well tissue culture plate seeded with 12500 capacitated or parental naive cells.

Figure S4 Application of SUSD2 for identification and purification of human naïve PSC during resetting and reprogramming, Relates to Figure 2 and Figure 4

(A) Flow cytometry analysis showing SUSD2 and CD24 expression of cell populations five days after sorting. SUSD2^{+}, SUSD2^{+}CD24^{-}; SUSD2^{-}, SUSD2^{-}CD24^{+}. Bright field and GFP images of cell populations at passage one and five after sorting. Scale bar, 50μm. (B) RT-qPCR analysis of general and naïve pluripotency markers in sorted SUSD2^{+}CD24^{-} and SUSD2^{-}CD24^{+} cells at P1, P3 and P10 after resetting. Error bars indicate s.d. of three biological replicates. (C) Flow cytometry analysis showing

SUSD2 and CD24 expression in cR-H9-EOS cells at passage 5 and 10. Left column shows unsorted reset cultures, bottom row shows cultures that were sorted for $SUSD2^{+}CD24^{-}$ at day 14 of resetting. (D) Images show bright field and immunofluorescence staining for SUSD2 on live cultures during episomal reprograming. Scale bar, 50µm. (E) Flow cytometry analysis showing SUSD2, EPCAM and CD24 expression during episomal reprograming. (F) Immunofluorescence staining for SUSD2 and KLF17 during episomal reprograming.

REFERENCES

Fusaki, N., Ban, H., Nishiyama, A., Saeki, K., and Hasegawa, M. (2009). Efficient induction of transgene-free human pluripotent stem cells using a vector based on Sendai virus, an RNA virus that does not integrate into the host genome. Proc Jpn Acad Ser B Phys Biol Sci *85*, 348-362.

Okita, K., Matsumura, Y., Sato, Y., Okada, A., Morizane, A., Okamoto, S., Hong, H., Nakagawa, M., Tanabe, K., Tezuka, K*., et al.* (2011). A more efficient method to generate integration-free human iPS cells. Nat Methods *8*, 409-412.

A

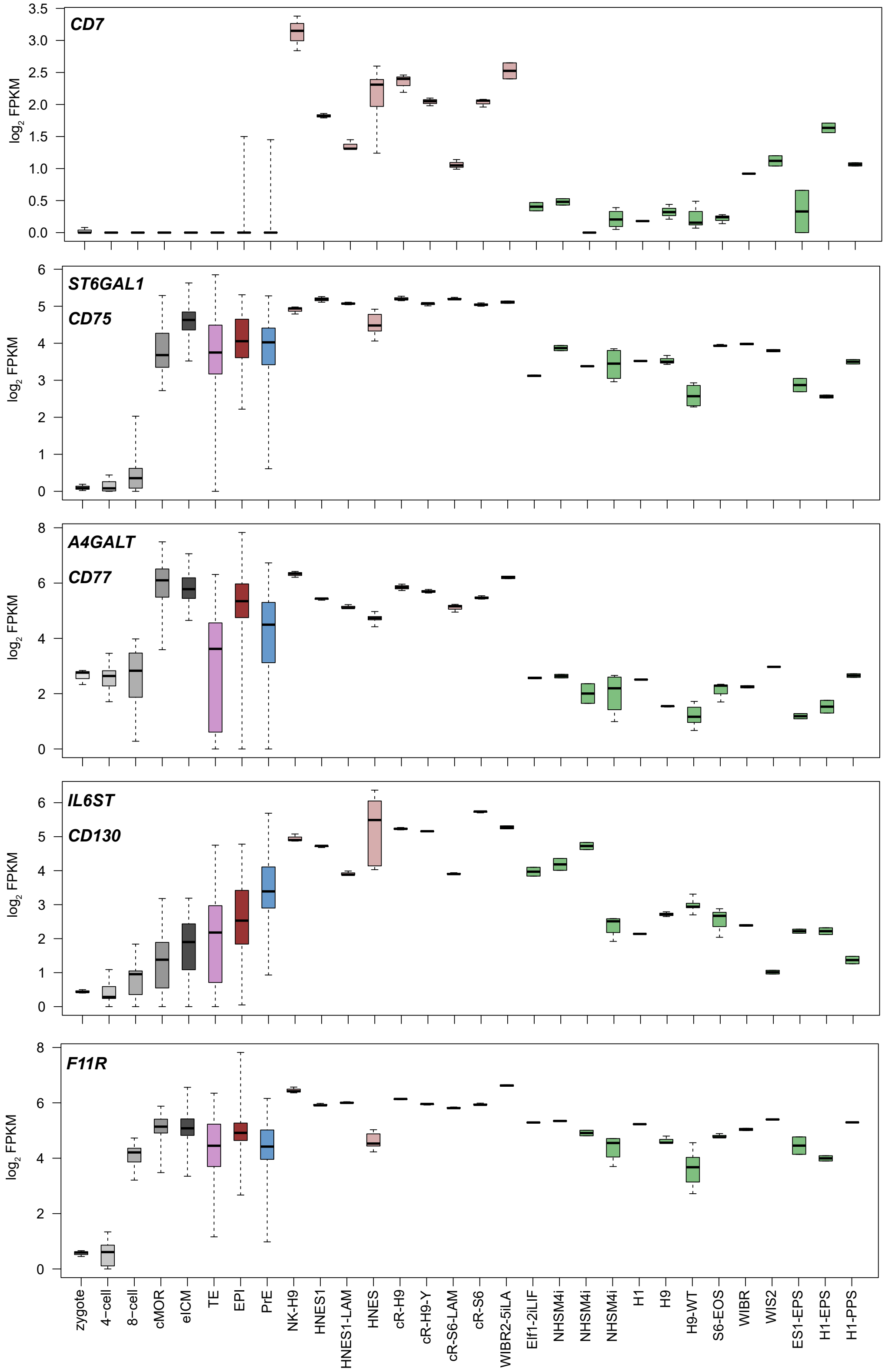

CD7
ST6GAL1
CD75
A4GALT
CD77
IL6ST
CD130
F11R
log2 FPKM
zygote
4−cell
8−cell
cMOR
eICM
TE
EPI
PrE
NK-H9
HNES1
HNES1-LAM
HNES
cR-H9
cR-H9-Y
cR-S6-LAM
cR-S6
WIBR2-5iLA
Elf1-2iLIF
NHSM4i
NHSM4i
NHSM4i
H1
H9
H9-WT
S6-EOS
WIBR
WIS2
ES1-EPS
H1-EPS
H1-PPS

B

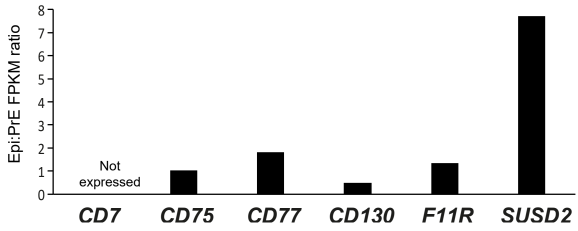

Epi:PrE FPKM ratio
Not expressed
CD7
CD75
CD77
CD130
F11R
SUSD2

Figure S1

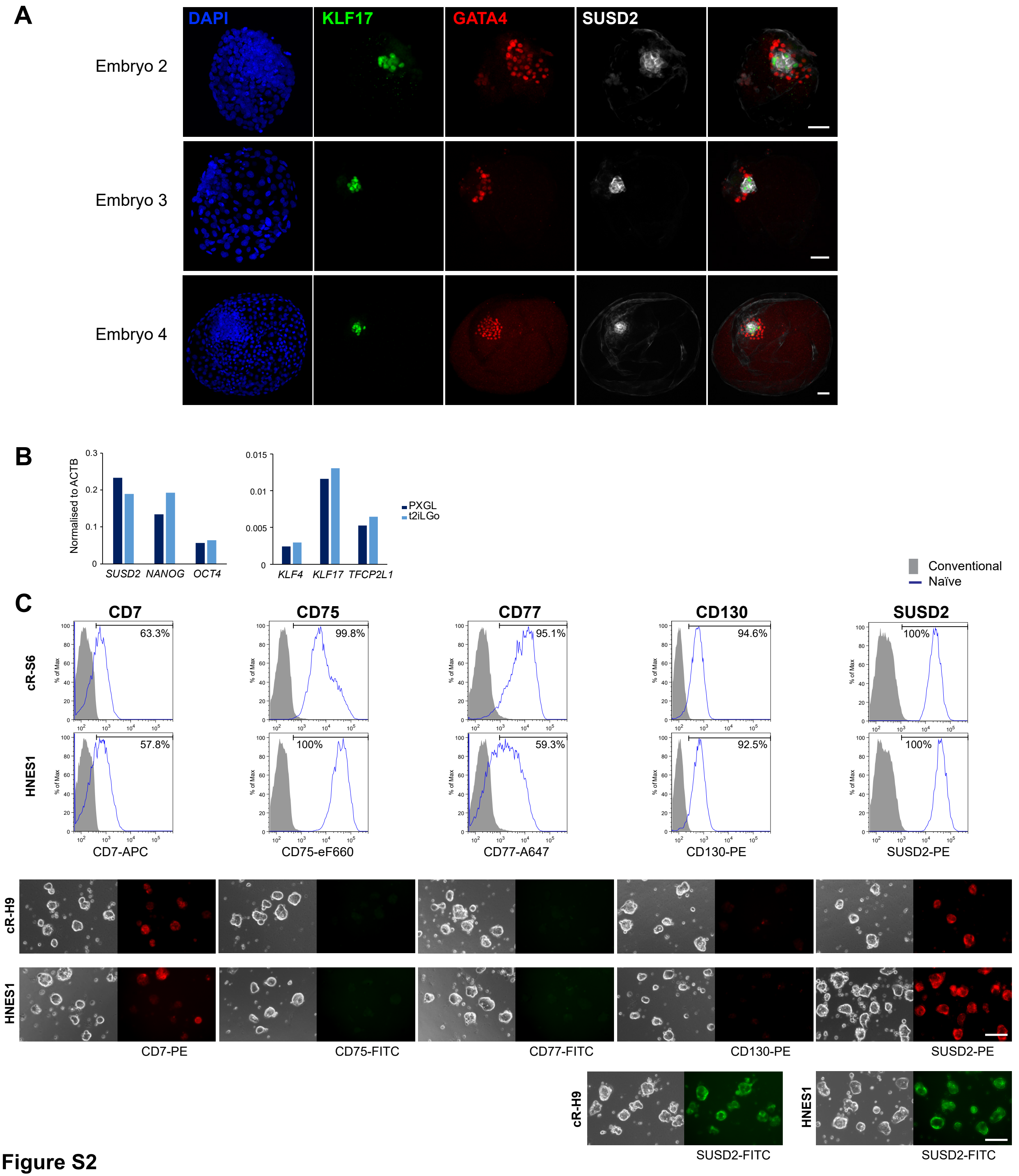
A
DAPI
KLF17
GATA4
SUSD2
Embryo 2
Embryo 3
Embryo 4
B
Normalised to ACTB
SUSD2
NANOG
OCT4
KLF4
KLF17
TFCP2L1
PXGL
t2iLGo
C
Conventional
Naïve
CD7
CD75
CD77
CD130
SUSD2
cR-S6
HNES1
63.3%
99.8%
95.1%
94.6%
100%
57.8%
100%
59.3%
92.5%
100%
% of Max
CD7-APC
CD75-eF660
CD77-A647
CD130-PE
SUSD2-PE
cR-H9
HNES1
CD7-PE
CD75-FITC
CD77-FITC
CD130-PE
SUSD2-PE
cR-H9
HNES1
SUSD2-FITC
SUSD2-FITC

Figure S2

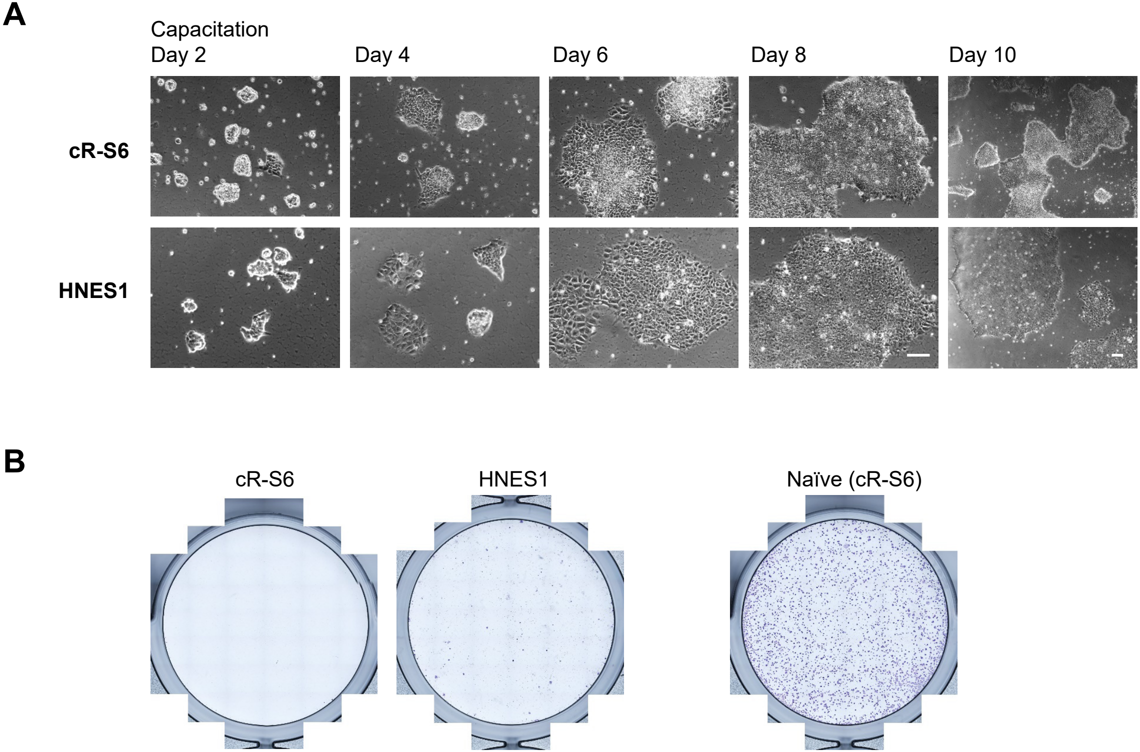

Capacitation for 10 days, replated to PXGL and AP stained 7 days later

Figure S3

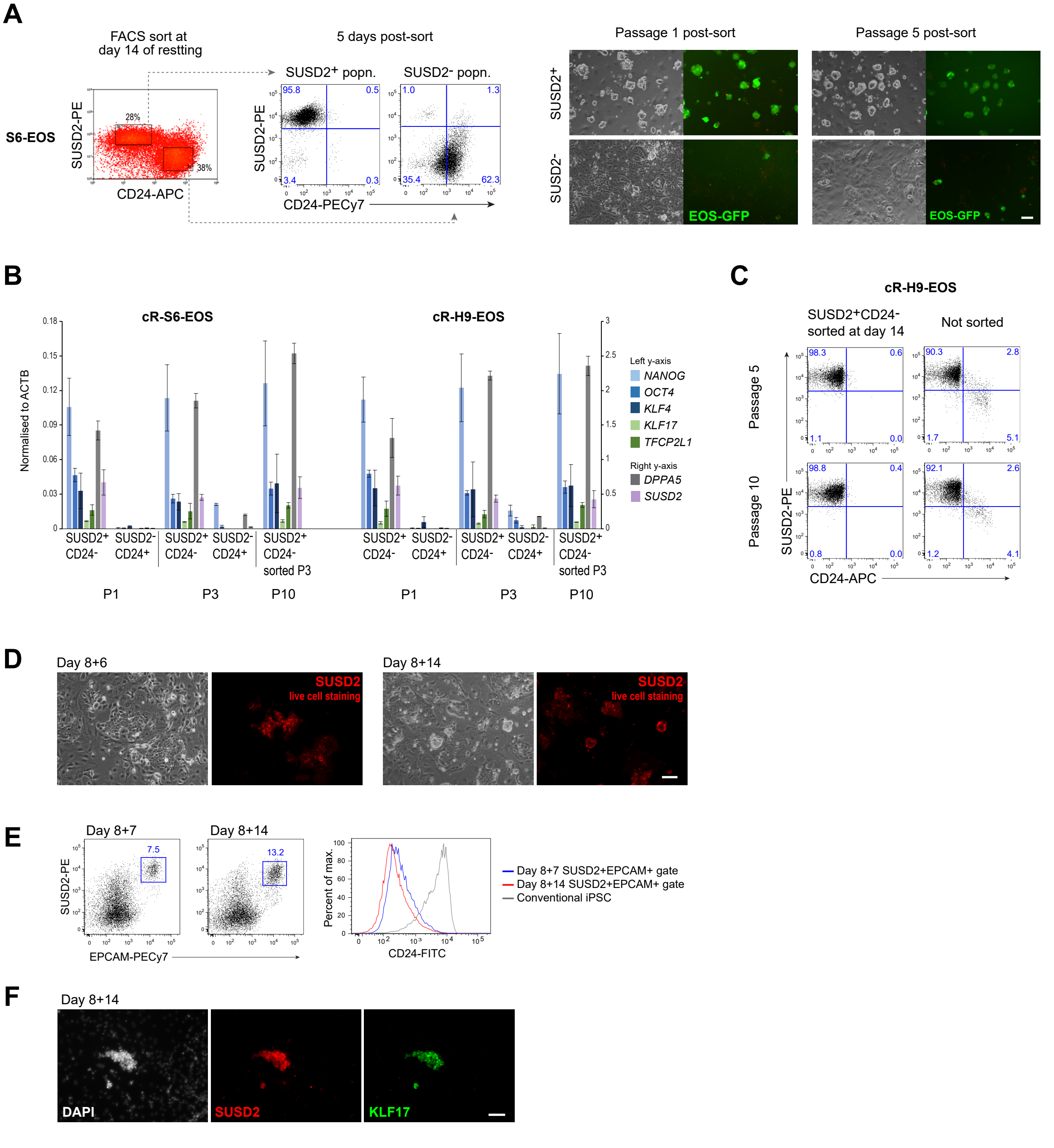
A
FACS sort at day 14 of restting
5 days post-sort
SUSD2+ popn.
SUSD2- popn.
S6-EOS
SUSD2-PE
CD24-APC
CD24-PECy7
Passage 1 post-sort
Passage 5 post-sort
SUSD2+
SUSD2-
EOS-GFP
B
cR-S6-EOS
cR-H9-EOS
Normalised to ACTB
Left y-axis
NANOG
OCT4
KLF4
KLF17
TFCP2L1
Right y-axis
DPPA5
SUSD2
SUSD2+ CD24-
SUSD2- CD24+
SUSD2+ CD24- sorted P3
P1
P3
P10
C
cR-H9-EOS
SUSD2+CD24- sorted at day 14
Not sorted
Passage 5
Passage 10
SUSD2-PE
CD24-APC
D
Day 8+6
Day 8+14
SUSD2 live cell staining
E
Day 8+7
Day 8+14
SUSD2-PE
EPCAM-PECy7
Percent of max.
CD24-FITC
Day 8+7 SUSD2+EPCAM+ gate
Day 8+14 SUSD2+EPCAM+ gate
Conventional iPSC
F
Day 8+14
DAPI
SUSD2
KLF17

Figure S4
