# Supplementary material for: The Cell-Surface Marker Sushi Containing Domain 2 Facilitates Establishment of Human Naive Pluripotent Stem Cells
Source: Stem Cell Reports. 2019 Apr 25;12(6):1212–22. doi: 10.1016/j.stemcr.2019.03.014 (PMC6565611; doi:10.1016/j.stemcr.2019.03.014)
Supplement: Document S2. Article plus Supplemental Information [file mmc2.pdf]

Stem Cell Reports
Report

OPEN ACCESS

The Cell-Surface Marker Sushi Containing Domain 2 Facilitates Establishment of Human Naive Pluripotent Stem Cells

Nicholas Bredenkamp,[1] Giuliano Giuseppe Stirparo,[1] Jennifer Nichols,[1,3] Austin Smith,[1,2,*] and Ge Guo[1,*]
[1]Wellcome–MRC Cambridge Stem Cell Institute, University of Cambridge, Cambridge CB2 1QR, UK
[2]Department of Biochemistry, University of Cambridge, Cambridge CB2 1GA, UK
[3]Department of Physiology, Development and Neuroscience, University of Cambridge, Cambridge CB2 3DY, UK
*Correspondence: austin.smith@cscr.cam.ac.uk (A.S.), gg251@cam.ac.uk (G.G.)
https://doi.org/10.1016/j.stemcr.2019.03.014

SUMMARY

Recently naive human pluripotent stem cells (hPSCs) have been described that relate to an earlier stage of development than conventional hPSCs. Naive hPSCs remain challenging to generate and authenticate, however. Here we report that Sushi Containing Domain 2 (SUSD2) is a robust cell-surface marker of naive hPSCs in the embryo and *in vitro*. *SUSD2* transcripts are enriched in the pre-implantation epiblast of human blastocysts and immunostaining shows localization of SUSD2 to KLF17-positive epiblast cells. *SUSD2* mRNA is strongly expressed in naive hPSCs but is negligible in other hPSCs. SUSD2 immunostaining of live or fixed cells provides unambiguous discrimination of naive versus conventional hPSCs. SUSD2 staining or flow cytometry enable monitoring of naive hPSCs in maintenance culture, and their isolation and quantification during resetting of conventional hPSCs or somatic cell reprogramming. Thus SUSD2 is a powerful non-invasive tool for reliable identification and purification of the naive hPSC phenotype.

INTRODUCTION

Pluripotent cells are present in the human embryo for around 10 days, from emergence in the blastocyst until lineage commitment during gastrulation (Rossant and Tam, 2017). *In vitro*, two classes of human pluripotent stem cell (hPSC) have been described. Conventional hPSCs, derived from inner cell mass (ICM) explants (O'Leary et al., 2012; Thomson et al., 1998) or generated by somatic cell reprogramming (induced pluripotent stem cells [(iPSCs]) (Takahashi et al., 2007), share characteristics with late post-implantation gastrulating epiblast (Nakamura et al., 2016). Propagation of stem cells resembling naive emergent epiblast has been reported more recently (Guo et al., 2017; Takashima et al., 2014; Theunissen et al., 2014). Naive hPSCs are generated by conversion from conventional PSCs, a process termed resetting. In addition, embryonic stem cells (ESCs) with naive features have been derived directly from dissociated ICM cells from day-6 human blastocysts (Guo et al., 2016). Naive-type hPSCs have also been obtained following somatic cell reprogramming (Kilens et al., 2018; Liu et al., 2017). The availability of naive and conventional hPSCs provides two complementary systems for modeling early human development. In particular, the global relationship of naive hPSCs to pre-implantation embryo epiblast (Nakamura et al., 2016; Stirparo et al., 2018) provides an opportunity to study and dissect the progression of pluripotency over a time window of human embryogenesis that is inaccessible *in utero*.

Current conditions employed to generate naive hPSCs show variation in efficiency, especially when applied across different cell lines (Guo et al., 2017). Reliable cell-surface markers would aid identification and establishment of naive hPSCs and facilitate optimization of culture conditions and procedures. To date several surface markers have been reported that may distinguish naive from conventional hPSCs. Some of these are expressed by conventional hPSCs and not by naive hPSCs (O'Brien et al., 2017; Pastor et al., 2016; Shakiba et al., 2015) while others show only differences in level between the two PSC types (Liu et al., 2017; O'Brien et al., 2017). On the other hand, markers claimed to be specific for naive PSCs (Collier et al., 2017) show either no expression or broad expression in early human embryos, challenging their relevance for identification specifically of the naive pluripotent phenotype.

Here we present Sushi Containing Domain 2 (SUSD2) (Sugahara et al., 2007) as a robust cell-surface marker of naive pluripotency in the human embryo and hPSCs *in vitro*. We identified high enrichment of *SUSD2* in naive pre-implantation epiblast through analysis of differential gene expression in human embryos. We evaluated SUSD2 protein expression by antibody staining of human blastocysts and of naive and conventional PSC cultures. Finally, we investigated the applicability of SUSD2 live cell staining and flow cytometry during resetting and reprogramming to naive PSC status.

RESULTS

Sushi Containing Domain 2 Is a Marker for Naive Pluripotency

To identify candidate markers for human naive pluripotent cells we scanned integrated single-cell RNA-sequencing

 © 2019 The Author(s).
This is an open access article under the CC BY license (http://creativecommons.org/licenses/by/4.0/).

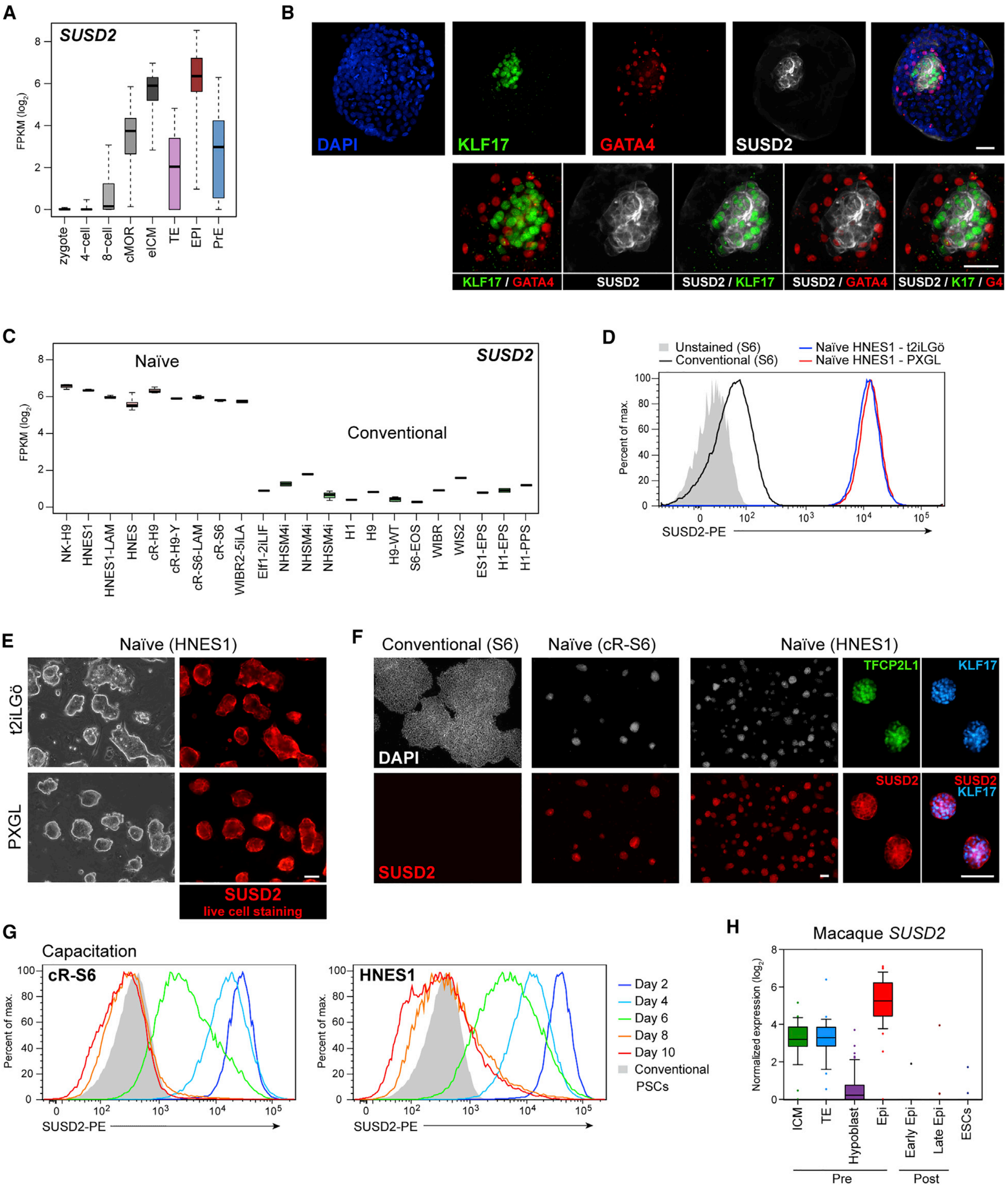

Figure 1. SUSD2 Is Expressed by Human Naive Pluripotent Cells in the Embryo and in Culture

(A) *SUSD2* transcript levels in human pre-implantation embryos at different stages and lineages, extracted from integrated single-cell RNA-sequencing data (Stirparo et al., 2018).

(B) Immunostaining for KLF17, GATA4, and SUSD2 in the E7 human blastocyst. Scale bars, 50 μm.

(C) *SUSD2* transcript levels in naive and conventional hPSCs (Stirparo et al., 2018).

(D) Flow-cytometry analysis of SUSD2 in conventional and naive cells.

(legend continued on next page)

datasets from early human embryos (Stirparo et al., 2018) for transmembrane proteins differentially expressed in the pre-implantation epiblast. We observed that Sushi Containing Domain 2 (*SUSD2*) is highly enriched in the ICM at embryonic day (E) 5 and has a mean level in epiblast cells at E6–7 that is 8-fold higher than in primitive endoderm or trophectoderm ($p < 0.005$) (Figure 1A). In contrast, other recently reported surface markers for naive cells are either not detectable or not specific to epiblast (Figure S1).

SUSD2 is a type I membrane protein with a large extracellular domain (Sugahara et al., 2007) against which there are several commercial antibodies (Sivasubramaniyan et al., 2013). We therefore examined whether SUSD2 protein expression reflects transcript distribution. We immunostained E7 human embryos using a monoclonal antibody. Intense cell-surface staining was observed on a subset of cells within the ICM (Figures 1B and S2A). These SUSD2 positive cells co-express the transcription factor KLF17, denoting human naive epiblast identity (Blakeley et al., 2015; Guo et al., 2016; Stirparo et al., 2018). In contrast, SUSD2 staining was faint in trophectoderm cells and absent in GATA4-positive hypoblast cells.

We then inspected publicly available hPSC transcriptome data (Gafni et al., 2013; Guo et al., 2016, 2017; Stirparo et al., 2018; Takashima et al., 2014; Theunissen et al., 2014; Ware et al., 2014; Yang et al., 2017). We found that *SUSD2* transcript levels are appreciable only in cells cultured in either t2iLGö or 5iLAF medium, which satisfy stringent criteria for naive pluripotent features (Davidson et al., 2015; Huang et al., 2014; Nakamura et al., 2016; Stirparo et al., 2018; Takashima et al., 2014; Theunissen et al., 2014, 2016) (Figure 1C). *SUSD2* mRNA is very low or absent in conventional or other hPSCs, including cultures in NHSM (Gafni et al., 2013) and so-called extended pluripotent stem cells (Yang et al., 2017). These observations indicate that SUSD2 expression may be a distinguishing marker for naive hPSCs.

We therefore investigated the utility of SUSD2 antibodies for discriminating hPSC phenotypes. Flow-cytometry analysis showed no detectable expression in conventional hPSC (Figure 1D). In contrast, SUSD2 was expressed unimodally at high levels in embryo-derived HNES1 naive hPSCs (Guo et al., 2016). This was the case both for cultures in the original t2iLGö formulation (Takashima et al., 2014), and in a modified version, PXGL (Guo et al., 2017), including the tankyrase inhibitor XAV939 and omitting GSK3 inhibition (for details see Experimental Procedures) (Figures 1D and S2B). SUSD2 was also highly expressed in chemically reset (cR) naive hPSCs in PXGL medium (Figure S2C). Comparative flow-cytometry analysis with other reported naive cell-surface markers (Collier et al., 2017) revealed that only CD75 exhibits a similar profile to SUSD2, while other markers did not effectively discriminate naive from conventional hPSCs, or were weakly expressed (Figure S2C).

We noted strong *in situ* cell-surface staining of naive hPSCs using a conjugated SUSD2 monoclonal antibody (Figure 1E). Importantly, live staining did not perturb cell viability or morphology, and naive cells could subsequently be expanded without consequence. With the exception of heterogeneous staining for CD7, *in situ* reactivity was not detected using conjugated antibodies for CD75 or other reported naive markers (Collier et al., 2017) (Figure S2C). We also evaluated SUSD2 immunostaining after paraformaldehyde fixation (Figure 1F). We detected no signal on conventional hPSCs but intense surface staining of naive cells. SUSD2-immunopositive cells co-expressed the naive transcription factor TFCP2L1 and the primate-specific naive transcription factor KLF17 (Blakeley et al., 2015; Guo et al., 2016; Takashima et al., 2014).

To initiate multi-lineage differentiation, naive hPSCs must transition to a state approaching conventional hPSCs, a process we have termed capacitation (Smith, 2017). Capacitation is achieved by withdrawal of t2iLGö or PXGL and culture for 8–10 days in N2B27 medium supplemented with XAV939 (Rostovskaya et al., 2019) (Figure S3A). SUSD2 expression is progressively downregulated during capacitation and is absent by day 8 (Figure 1G). Change in cell state after capacitation is confirmed by loss of colony-forming ability in naive cell medium (Figure S3B). We have shown elsewhere that naive hPSC capacitation reflects *in vivo* progression of the epiblast from ICM to late post-implantation in the embryo of the non-human primate *Macaca fascicularis* (Rostovskaya et al., 2019). We examined the *Macaca* dataset (Nakamura et al., 2016) and found *SUSD2* mRNA is present in pre-implantation epiblast but not at post-implantation stages (Figure 1H). Thus, SUSD2 expression in hPSCs is closely linked to naive status both in cultured stem cells and in the primate embryo.

(E) Images of bright-field and SUSD2 immunostaining using a SUSD2-PE antibody. Scale bar, 50 μm.
(F) Immunostaining for SUSD2, TFCP2L1, and KLF17 in conventional and naive (cR-S6 and HNES1) cells. Scale bars, 100 μm.
(G) Flow-cytometry analysis of SUSD2 expression during capacitation of cR-S6 and HNES1 cells.
(H) *SUSD2* transcript levels in *Macaca fascicularis* embryos (Nakamura et al., 2016). cMOR, compacted morula; eICM, early inner cell mass; TE, trophectoderm; Epi, epiblast; PrE, primitive endoderm.
See also Figures S1–S3.

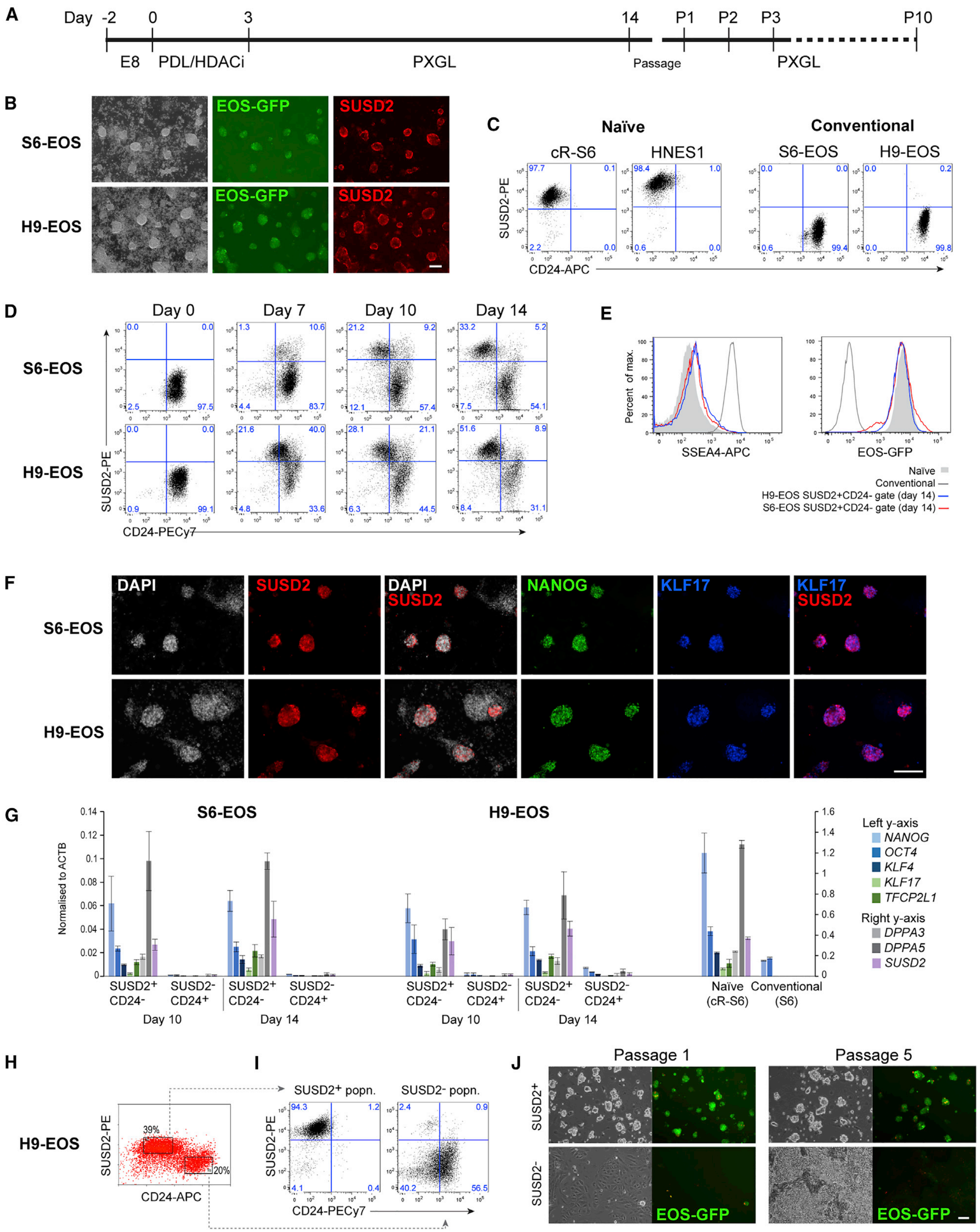

(legend on next page)

SUSD2 Identifies Naive hPSCs after Chemical Resetting

Conventional hPSCs can be reset to naive status by short-term exposure to the histone deacetylase inhibitor valproic acid followed by culture in naive medium (Guo et al., 2017). Naive hPSCs are then enriched by continuous passaging. We investigated whether SUSD2 could be utilized to identify naive cells upon chemical resetting (Figure 2A). We used Shef6 (S6)-EOS and H9-EOS hPSCs in which the *PB-EOS-GFP* transgene provides a reporter of naive status (Guo et al., 2017; Takashima et al., 2014). Live cell imaging on day 10 of resetting revealed co-expression of EOS-GFP and SUSD2 in emerging domed naive-type colonies (Figure 2B).

We performed flow-cytometry analyses for SUSD2 along with the conventional hPSC marker, CD24 (Shakiba et al., 2015). SUSD2$^+$CD24$^-$ and SUSD2$^-$CD24$^+$ cell-surface phenotypes unambiguously distinguish naive from conventional hPSCs (Figure 2C). At the start of resetting, all hPSCs are SUSD2$^-$CD24$^+$. By day 10, a substantial proportion (20%–30%) of cells are SUSD2$^+$ and either CD24 low or CD24 negative (Figure 2D). By day 14, most SUSD2$^+$ cells are negative for CD24 and also for SSEA4 (Figures 2D and 2E), another marker of conventional PSCs that is absent on naive hPSCs (Pastor et al., 2016). The vast majority of SUSD2$^+$ (>95%) cells at this stage express EOS-GFP (Figure 2E).

Immunostaining of S6-EOS and H9-EOS cultures at day 14 of resetting showed co-expression of SUSD2 with transcription factors KLF17 and NANOG (Figure 2F). SUSD2-negative cells did not express either of these factors. We purified SUSD2$^+$CD24$^-$ populations at days 10 and 14 for further analysis of marker expression by qRT-PCR. Expression of naive markers *KLF4*, *KLF17*, *TFCP2L1*, *DPPA3*, and *DPPA5* was restricted to SUSD2$^+$CD24$^-$ cells and absent or very low in the SUSD2$^-$CD24$^+$ fraction (Figure 2G).

Together, these observations indicate that SUSD2 staining in combination with absence of CD24 or SSEA4 identifies reset naive PSCs. We therefore investigated whether SUSD2 antibody staining could be utilized to purify naive PSCs from resetting cultures. We fractionated SUSD2$^+$CD24$^-$ and SUSD2$^-$CD24$^+$ populations by flow cytometry on day 14 of resetting and plated them in PXGL (Figures 2H and S4A). Analysis 5 days later showed that the great majority of cells retained the cell-surface phenotype from sorting (Figures 2I and S4A). Consistent with the flow profile, SUSD2$^+$CD24$^-$ sorted cells generated numerous EOS-GFP$^+$ dome-shaped colonies with few if any other colony types apparent. In contrast, SUSD2$^-$CD24$^+$ cells yielded predominantly heterogeneously differentiated cells that were EOS-GFP$^-$, with only the occasional GFP$^+$ domed colony (Figures 2J and S4A).

The SUSD2$^+$CD24$^-$ sorted cultures retained morphological naive features and maintained EOS-GFP expression upon passaging (Figures 2J and S4A). qRT-PCR analysis at passage 1 (P1) and P3 after sorting showed that expression of naive markers *KLF4*, *KLF17*, *TFCP2L1*, and *DPPA5* was sustained in SUSD2$^+$CD24$^-$-derived cells but remained negligible in the SUSD2$^-$CD24$^+$ derivatives (Figure S4B). Flow-cytometry analysis of SUSD2$^+$CD24$^-$ sorted cells at later passages showed maintenance of purified SUSD2$^+$ cells (>98%). In contrast, parallel unsorted reset cultures retained subpopulations of both SUSD2^{lo}CD24$^+$ and SUSD2$^-$CD24$^+$ cells, in addition to the major SUSD2$^+$CD24$^-$ population (Figure S4C).

SUSD2 Sorting Facilitates Establishment of Naive hPSC Cultures

The efficiency of chemical or transgene-driven resetting from conventional to naive hPSCs is variable, and extended passaging may be required to establish homogeneous cultures (Guo et al., 2017; Takashima et al., 2014). We utilized SUSD2 to monitor efficiency and purify naive cells during resetting of diverse human ESC and iPSC lines. We applied the chemical resetting protocol to two conventional human ESCs, H1 and H7 (Thomson et al., 1998) and two iPSCs, MeCP2-clone17 and NCRM-2. Flow-cytometry analysis of resetting cultures at day 10, day 14, P1, and P3

Figure 2. SUSD2 Identifies and Purifies Reset Naive hPSCs

(A) Schematic of the chemical resetting protocol. HDACi, histone deacetylase inhibitor.
(B) Images of cultures at day 10 of resetting. Scale bar, 50 μm.
(C and D) Flow-cytometry analysis of SUSD2 and CD24 expression in conventional and naive hPSCs (C) and during resetting (D).
(E) Flow-cytometry analysis of GFP and SSEA4 on SUSD2$^+$CD24$^-$ cells at day 14 of resetting.
(F) Immunostaining for SUSD2, NANOG, and KLF17 at day 14 of resetting. Scale bar, 100 μm.
(G) qRT-PCR analysis of sorted SUSD2$^+$CD24$^-$ and SUSD2$^-$CD24$^+$ cells at day 10 and day 14 of resetting. Error bars indicate SD of three independent experiments.
(H) Flow cytometry sort plot at day 14 of resetting.
(I) Flow-cytometry analysis of SUSD2 and CD24 expression on cell populations sorted in (H), 5 days after sorting. SUSD2$^+$, SUSD2$^+$CD24$^-$; SUSD2$^-$, SUSD2$^-$CD24$^+$.
(J) Bright-field and GFP images of cell populations sorted in (H) at passages 1 and 5 after sorting. Scale bar, 50 μm.
See also Figure S4.

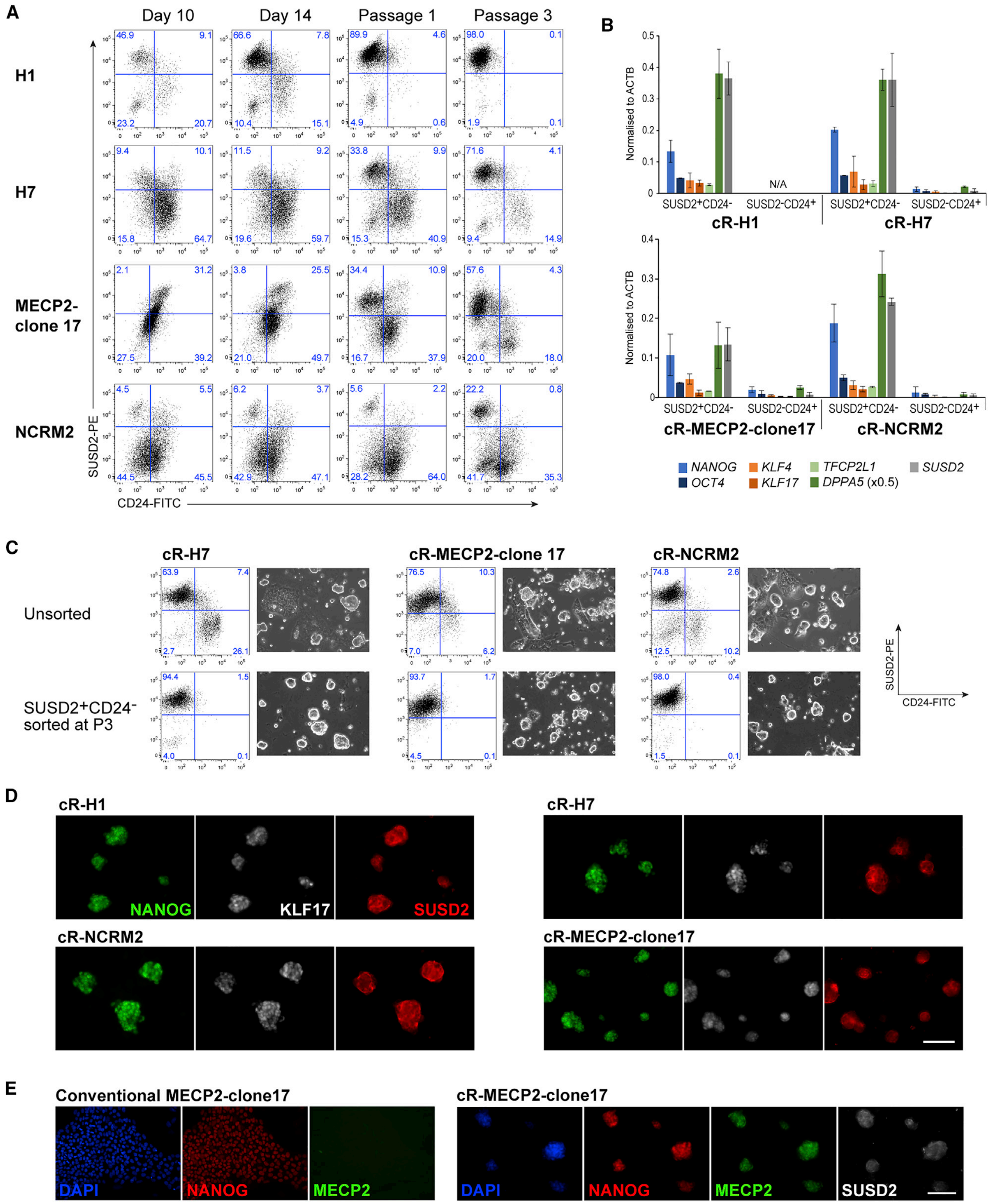

Figure 3. SUSD2 Identifies Reset Naive hPSCs upon Resetting of Multiple Cell Lines

(A) Flow-cytometry analysis of SUSD2 and CD24 expression during resetting of two human ESC lines (H1 and H7) and two iPSC lines (MECP2-clone17 and NCRM-2).

(legend continued on next page)

revealed that SUSD2$^+$CD24$^-$ populations appeared with different frequencies and kinetics for the individual cell lines (Figure 3A). Resetting efficiency, quantified by SUSD2$^+$CD24$^-$ cell fraction at P3, ranged from 22% to 98% (Figure 3A). The identity of SUSD2$^+$ cells was evaluated by qRT-PCR analysis whereby, as shown previously, expression of naive markers *KLF4*, *KLF17*, *TFCP2L1*, and *DPPA5* was restricted to SUSD2$^+$CD24$^-$ cells (Figure 3B).

We purified SUSD2$^+$CD24$^-$ cells by flow cytometry at P3. Replating resulted in uniform cultures of naive-type colonies. In contrast, unsorted cultures displayed persisting heterogeneity (Figure 3C). Immunostaining showed co-expression of SUSD2, NANOG, and KLF17 throughout naive cultures after sorting (Figure 3D). Post-sort cultures were expanded for ten passages in PXGL and retained domed colony morphology.

MeCP2-clone17 cells are heterozygous for a loss-of-function mutation in the X-linked gene *MECP2* (Lee et al., 2001; Sahakyan et al., 2017). MeCP2 protein is not expressed in conventional MeCP2-clone17 cells because the wild-type allele is on the silent X chromosome. Upon resetting, the silent X is expected to be reactivated (Guo et al., 2017). Consistent with this, immunostaining revealed co-expression of MeCP2 with SUSD2 in sorted reset cultures, providing further evidence of naive status (Figure 3E).

These findings demonstrate the utility of SUSD2$^+$ staining during chemical resetting, bypassing the requirement for a transgenic reporter or prolonged passaging to establish homogeneous naive cultures from different starting hPSC lines, even when the initial frequency of resetting is poor.

SUSD2 Identifies Emerging Naive hPSCs during Somatic Cell Reprogramming

Naive hPSCs can be generated from somatic cells by molecular reprogramming (Giulitti et al., 2018; Kilens et al., 2018; Liu et al., 2017). We used Sendai viral (SeV) vectors to reprogram human diploid fibroblasts (Figure 4A) (Fusaki et al., 2009). One week after transfection we observed small patches of cells undergoing mesenchymal-to-epithelial transition. On day 8 culture medium was exchanged to PXGL supplemented with ROCK inhibitor Y-27632 (Figure 4A). Six days following transfer to PXGL, SUSD2-positive cells emerged (Figure 4B). By 10 days multiple SUSD2-positive domed colonies were apparent. Flow-cytometry analysis showed that the majority of cells in the culture at day 10 are SUSD2$^+$ and CD24$^-$ (Figure 4C). These cultures can be bulk passaged and further propagated to establish naive iPSCs.

We also examined SUSD2 expression during reprogramming using episomal *pCXLE* vectors (Okita et al., 2011) and found that positive cells appeared with similar kinetics as in SeV reprogramming. However, the episomal system produced more heterogeneous cultures (Figure S4D). We therefore used EpCAM to exclude any mesenchymal cells that may express SUSD2 (Sivasubramaniyan et al., 2013). Flow-cytometry analysis identified an EpCAM$^+$SUSD2$^+$CD24$^-$ population 7 days after transfer to PXGL that increased to 13% of the culture by day 14 (Figure S4E). Immunostaining of reprogrammed cultures on day 14 showed co-expression of SUSD2 with KLF17 in both SeV and episomal systems (Figures 4D and S4F). qRT-PCR analysis of isolated SUSD2$^+$CD24$^-$ populations in both cases confirmed expression of naive pluripotency markers at similar levels to embryo-derived HNES1 naive hPSCs (Figure 4E). These results demonstrate that SUSD2 marks emerging naive iPSCs during somatic cell reprogramming.

DISCUSSION

SUSD2 is a type I membrane protein of unknown function (Sugahara et al., 2007). It is expressed by various cell types in development and adulthood, and has been used to fractionate both pancreatic and mesenchymal progenitors (Masuda et al., 2012; Ramond et al., 2017; Sivasubramaniyan et al., 2013). However, it has not previously been associated with pluripotent cells, *in vivo* or *in vitro*.

Our findings reveal that SUSD2 is highly enriched in naive epiblast in the human blastocyst and uniformly expressed on the cell surface of naive hPSCs, but is absent from other hPSCs. SUSD2 is lost as naive hPSCs transition toward differentiation, consistent with transcriptional downregulation observed in the post-implantation cynomolgus embryo. Future investigation will determine whether the restricted expression of SUSD2 has functional consequence for naive pluripotency.

SUSD2 antibody binding provides non-invasive labeling for live cell imaging and flow-cytometric cell sorting with no evident deleterious effects. SUSD2 strongly stains naive

(B) qRT-PCR analysis of marker expression in sorted SUSD2$^+$CD24$^-$ and SUSD2$^-$CD24$^+$ cells at passage 3 (P3). Note that no SUSD2$^-$CD24$^+$ population is evident for the cR-H1 line. *DPPA5* expression is shown at 0.5× actual expression. Error bars indicate SD of two independent experiments.
(C) Flow-cytometry analysis of SUSD2 and CD24 expression and bright-field images of reset cultures at P5. Top row: unsorted reset cultures; bottom row: cultures sorted for SUSD2$^+$CD24$^-$ at P3. Scale bar, 50 μm.
(D) Immunostaining for SUSD2, NANOG, and KLF17 at P6 for cultures that were sorted for SUSD2$^+$CD24$^-$ at P3. Scale bar, 100 μm.
(E) Immunostaining for NANOG, MECP2, and SUSD2 on reset (P6) and parental MECP2-clone17 cells. Scale bar, 100 μm.

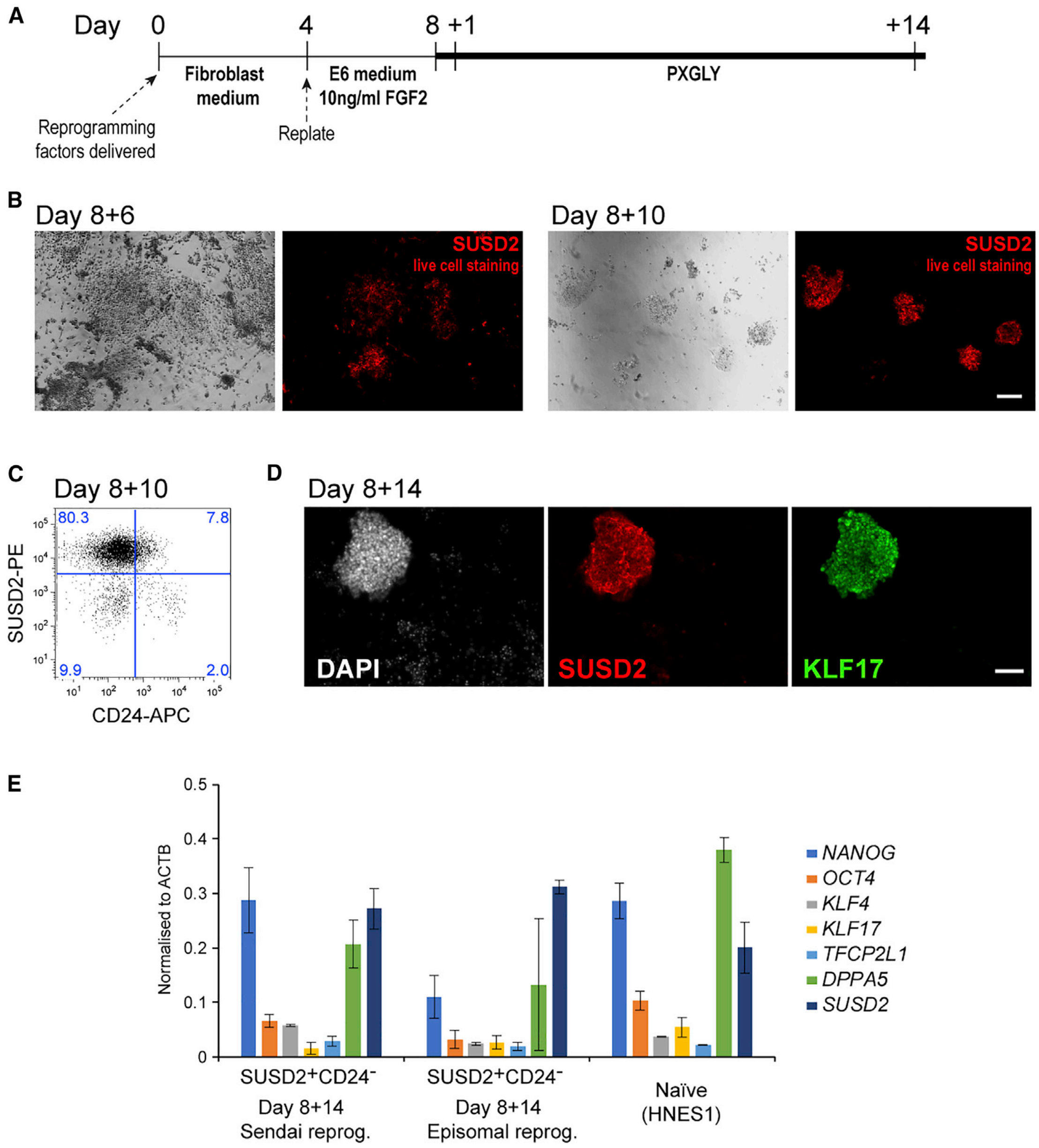

Figure 4. SUSD2 Identifies Naive hPSCs during Somatic Cell Reprogramming
(A) Schematic of the reprogramming protocol.
(B–D) Sendai vector reprogramming of human dermal fibroblasts. (B) Images of bright-field and SUSD2 live staining. Scale bar, 50 μm. (C) Flow-cytometry analysis of SUSD2 and CD24 expression. (D) Immunostaining for SUSD2, NANOG, and KLF17. Scale bar, 50 μm.
(E) qRT-PCR analysis of markers on sorted SUSD2$^+$CD24$^-$ at day 8 + 14 for Sendai vector- and episomal-mediated reprogramming, and on established naive hPSCs. Bars indicate the SD of two independent experiments.
See also Figure S4.

hPSCs *in situ* and allows unambiguous discrimination of naive from conventional hPSCs by flow cytometry. Several candidate markers for distinguishing naive hPSCs have recently been reported (Collier et al., 2017; Liu et al., 2017; O'Brien et al., 2017; Pastor et al., 2016; Shakiba et al., 2015) and reviewed (Trusler et al., 2018). Among these, F11R is highly expressed by naive hPSCs (O'Brien et al., 2017) but is also present at substantial levels on conventional cells (Liu et al., 2017) (Figure S1). Of the other markers reported for naive hPSCs (Collier et al., 2017), CD75, CD77, and CD130 display broad mRNA expression and antibody staining in E6 embryos while CD7 mRNA is

not detected in the human embryo. CD7 and CD77 do not resolve naive hPSCs from conventional hPSCs by flow cytometry, while CD130 expression is weak. CD75, CD77, and CD130 antibodies did not stain naive hPSCs *in situ* while anti-CD7 stained heterogeneously. In contrast, SUSD2 decisively labels naive hPSCs, and provides assurance of embryo lineage and hPSC classification not offered by current markers.

Live cell staining or flow-cytometry analysis for SUSD2 provides simple and reliable means to routinely monitor hPSC culture status. This is useful because culture conditions for naive hPSCs have yet to be fully optimized, and cells can become heterogeneous during maintenance. SUSD2 is particularly valuable in facilitating the establishment of naive hPSCs by resetting or reprogramming procedures. Current protocols are relatively inefficient, and extended passaging may be required to enrich for the naive phenotype. Sorting for SUSD2$^+$ and against a conventional hPSC marker such as CD24 or SSEA4 allows efficient purification from mixed resetting cultures and, thus, more rapid establishment of naive PSC lines.

In summary, our findings illustrate the utility of SUSD2 antibody staining for classification, quantification, and isolation of naive hPSCs. We envisage that SUSD2 staining will be a useful tool for optimization of resetting and reprogramming conditions. It will also be of future interest to determine whether SUSD2 plays a significant biological role in naive pluripotent cells.

EXPERIMENTAL PROCEDURES

Ethics Statement

Human embryo research was licensed by the UK Human Fertilization and Embryology Authority under research licence RO178. Supernumerary embryos were donated from *in vitro* fertilization programs with informed consent.

Cell Culture

Conventional hPSC cultures were propagated on Geltrex (growth factor-reduced, Thermo Fisher, A1413302) in Essential 8 (E8) medium made in-house (Chen et al., 2011). hESC lines were Shef6, S6-EOS, H9-EOS, H1, and H7; human iPSCs were NCRM-2 (NINDS Repository) and MECP2-clone17 (Sahakyan et al., 2017) gifted by Kathrin Plath. Chemically reset (cR) or embryo-derived (HNES1) naive PSCs were propagated in N2B27 with PXGL. Cells were cultured in 5% O_2, 7% CO_2 in a humidified incubator at 37°C and passaged by dissociation with Accutase (Thermo Fisher Scientific, A1110501) every 3–5 days. Cell lines were confirmed free of mycoplasma contamination by periodic in-house PCR assay. For capacitation, cells were passaged once without feeders in PXGL medium and then exchanged into N2B27 containing 2 μM XAV939 (Rostovskaya et al., 2019). Chemical resetting was performed as described by Guo et al. (2017) with minor modifications. Somatic cell reprogramming was performed either with Sendai virus vectors (Fusaki et al., 2009) or with episomal vectors (*pCXLE-OCT4-shRNA*(*p53*), *pCXLE-SOX2-KLF4*, and *pCXLE-L-MYC-LIN28* [Okita et al., 2011]). For detailed procedures, see Supplemental Experimental Procedures.

Human Embryo Culture and Staining

Frozen embryos were thawed at day 5 or 6 post fertilization directly into N2B27 and cultured until fixation at day 7 post fertilization. Blastocysts were fixed in 4% PFA, immunostained, and imaged as described (Takashima et al., 2014).

SUPPLEMENTAL INFORMATION

Supplemental Information can be found online at https://doi.org/10.1016/j.stemcr.2019.03.014.

AUTHOR CONTRIBUTIONS

Conceptualization, N.B., G.G., and A.S.; Investigation, N.B., G.G., and J.N.; Methodology, N.B. and G.G.; Formal analysis, G.G.S.; Writing, A.S., N.B., and G.G.; Supervision, G.G. and A.S.

ACKNOWLEDGMENTS

We thank James Clarke and Rosalind Drummond for technical support. Andy Riddell, Peter Humphreys, and Darran Clements supported flow cytometry and imaging studies. Elizabeth Apsley assisted with the episomal reprogramming experiments. Maria Rostovskaya developed the capacitation protocol. We are most grateful to patients who donated human embryos and to clinical embryologists and nursing staff at Nuffield Health Woking Hospital who facilitated these donations. This research was funded by the Medical Research Council of the United Kingdom (G1001028 and MR/P00072X/1) and European Commission Framework 7 (HEALTH-F4-2013-602423, PluriMes). The Cambridge Stem Cell Institute receives core support from the Wellcome Trust and the Medical Research Council. A.S. is a Medical Research Council Professor.

Received: November 3, 2018
Revised: March 28, 2019
Accepted: March 29, 2019
Published: April 25, 2019

REFERENCES

Blakeley, P., Fogarty, N.M.E., del Valle, I., Wamaitha, S.E., Hu, T.X., Elder, K., Snell, P., Christie, L., Robson, P., and Niakan, K.K. (2015). Defining the three cell lineages of the human blastocyst by single-cell RNA-seq. Development *142*, 3151–3165.

Chen, G., Gulbranson, D.R., Hou, Z., Bolin, J.M., Ruotti, V., Probasco, M.D., Smuga-Otto, K., Howden, S.E., Diol, N.R., Propson, N.E., et al. (2011). Chemically defined conditions for human iPSC derivation and culture. Nat. Methods *8*, 424–429.

Collier, A.J., Panula, S.P., Schell, J.P., Chovanec, P., Plaza Reyes, A., Petropoulos, S., Corcoran, A.E., Walker, R., Douagi, I., Lanner, F., et al. (2017). Comprehensive cell surface protein profiling identifies specific markers of human naive and primed Pluripotent states. Cell Stem Cell *20*, 874–890.e7.

Davidson, K.C., Mason, E.A., and Pera, M.F. (2015). The pluripotent state in mouse and human. Development *142*, 3090–3099.

Fusaki, N., Ban, H., Nishiyama, A., Saeki, K., and Hasegawa, M. (2009). Efficient induction of transgene-free human pluripotent stem cells using a vector based on Sendai virus, an RNA virus that does not integrate into the host genome. Proc. Jpn. Acad. Ser. B Phys. Biol. Sci. *85*, 348–362.

Gafni, O., Weinberger, L., Mansour, A.A., Manor, Y.S., Chomsky, E., Ben-Yosef, D., Kalma, Y., Viukov, S., Maza, I., Zviran, A., et al. (2013). Derivation of novel human ground state naive pluripotent stem cells. Nature *504*, 282–286.

Giulitti, S., Pellegrini, M., Zorzan, I., Martini, P., Gagliano, O., Mutarelli, M., Ziller, M.J., Cacchiarelli, D., Romualdi, C., Elvassore, N., et al. (2018). Direct generation of human naive induced pluripotent stem cells from somatic cells in microfluidics. Nat. Cell Biol. *21*, 275–286.

Guo, G., von Meyenn, F., Rostovskaya, M., Clarke, J., Dietmann, S., Baker, D., Sahakyan, A., Myers, S., Bertone, P., Reik, W., et al. (2017). Epigenetic resetting of human pluripotency. Development *144*, 2748–2763.

Guo, G., von Meyenn, F., Santos, F., Chen, Y., Reik, W., Bertone, P., Smith, A., and Nichols, J. (2016). Naive pluripotent stem cells derived directly from isolated cells of the human inner cell mass. Stem Cell Reports *6*, 437–446.

Huang, K., Maruyama, T., and Fan, G. (2014). The naive state of human pluripotent stem cells: a synthesis of stem cell and preimplantation embryo transcriptome analyses. Cell Stem Cell *15*, 410–415.

Kilens, S., Meistermann, D., Moreno, D., Chariau, C., Gaignerie, A., Reignier, A., Lelièvre, Y., Casanova, M., Vallot, C., Nedellec, S., et al. (2018). Parallel derivation of isogenic human primed and naive induced pluripotent stem cells. Nat. Commun. *9*, 360.

Lee, S.S., Wan, M., and Francke, U. (2001). Spectrum of MECP2 mutations in Rett syndrome. Brain Dev. *23* (*Suppl 1*), S138–S143.

Liu, X., Nefzger, C.M., Rossello, F.J., Chen, J., Knaupp, A.S., Firas, J., Ford, E., Pflueger, J., Paynter, J.M., Chy, H.S., et al. (2017). Comprehensive characterization of distinct states of human naive pluripotency generated by reprogramming. Nat. Methods *14*, 1055–1062.

Masuda, H., Anwar, S.S., Buhring, H.J., Rao, J.R., and Gargett, C.E. (2012). A novel marker of human endometrial mesenchymal stem-like cells. Cell Transpl. *21*, 2201–2214.

Nakamura, T., Okamoto, I., Sasaki, K., Yabuta, Y., Iwatani, C., Tsuchiya, H., Seita, Y., Nakamura, S., Yamamoto, T., and Saitou, M. (2016). A developmental coordinate of pluripotency among mice, monkeys and humans. Nature *537*, 57–62.

O'Brien, C.M., Chy, H.S., Zhou, Q., Blumenfeld, S., Lambshead, J.W., Liu, X., Kie, J., Capaldo, B.D., Chung, T.L., Adams, T.E., et al. (2017). New monoclonal antibodies to defined cell surface proteins on human pluripotent stem cells. Stem Cells *35*, 626–640.

O'Leary, T., Heindryckx, B., Lierman, S., van Bruggen, D., Goeman, J.J., Vandewoestyne, M., Deforce, D., de Sousa Lopes, S.M., and De Sutter, P. (2012). Tracking the progression of the human inner cell mass during embryonic stem cell derivation. Nat. Biotechnol. *30*, 278–282.

Okita, K., Matsumura, Y., Sato, Y., Okada, A., Morizane, A., Okamoto, S., Hong, H., Nakagawa, M., Tanabe, K., Tezuka, K., et al. (2011). A more efficient method to generate integration-free human iPS cells. Nat. Methods *8*, 409–412.

Pastor, W.A., Chen, D., Liu, W., Kim, R., Sahakyan, A., Lukianchikov, A., Plath, K., Jacobsen, S.E., and Clark, A.T. (2016). Naive human pluripotent cells feature a methylation landscape devoid of blastocyst or germline memory. Cell Stem Cell *18*, 323–329.

Ramond, C., Glaser, N., Berthault, C., Ameri, J., Kirkegaard, J.S., Hansson, M., Honore, C., Semb, H., and Scharfmann, R. (2017). Reconstructing human pancreatic differentiation by mapping specific cell populations during development. Elife *6*. https://doi.org/10.7554/eLife.27564.

Rossant, J., and Tam, P.P.L. (2017). New insights into early human development: lessons for stem cell derivation and differentiation. Cell Stem Cell *20*, 18–28.

Rostovskaya, M., Stirparo, G.G., and Smith, A. (2019). Capacitation of human naive pluripotent stem cells for multi-lineage differentiation. Development *146*. https://doi.org/10.17863/CAM.37544.

Sahakyan, A., Kim, R., Chronis, C., Sabri, S., Bonora, G., Theunissen, T.W., Kuoy, E., Langerman, J., Clark, A.T., Jaenisch, R., et al. (2017). Human naive pluripotent stem cells model X chromosome dampening and X inactivation. Cell Stem Cell *20*, 87–101.

Shakiba, N., White, C.A., Lipsitz, Y.Y., Yachie-Kinoshita, A., Tonge, P.D., Hussein, S.M., Puri, M.C., Elbaz, J., Morrissey-Scoot, J., Li, M., et al. (2015). CD24 tracks divergent pluripotent states in mouse and human cells. Nat. Commun. *6*, 7329.

Sivasubramaniyan, K., Harichandan, A., Schumann, S., Sobiesiak, M., Lengerke, C., Maurer, A., Kalbacher, H., and Buhring, H.J. (2013). Prospective isolation of mesenchymal stem cells from human bone marrow using novel antibodies directed against Sushi domain containing 2. Stem Cells Dev. *22*, 1944–1954.

Smith, A. (2017). Formative pluripotency: the executive phase in a developmental continuum. Development *144*, 365–373.

Stirparo, G.G., Boroviak, T., Guo, G., Nichols, J., Smith, A., and Bertone, P. (2018). Integrated analysis of single-cell embryo data yields a unified transcriptome signature for the human pre-implantation epiblast. Development *145*. https://doi.org/10.1242/dev.158501.

Sugahara, T., Yamashita, Y., Shinomi, M., Yamanoha, B., Iseki, H., Takeda, A., Okazaki, Y., Hayashizaki, Y., Kawai, K., Suemizu, H., et al. (2007). Isolation of a novel mouse gene, mSVS-1/SUSD2, reversing tumorigenic phenotypes of cancer cells in vitro. Cancer Sci. *98*, 900–908.

Takahashi, K., Tanabe, K., Ohnuki, M., Narita, M., Ichisaka, T., Tomoda, K., and Yamanaka, S. (2007). Induction of pluripotent stem cells from adult human fibroblasts by defined factors. Cell *131*, 861–872.

Takashima, Y., Guo, G., Loos, R., Nichols, J., Ficz, G., Krueger, F., Oxley, D., Santos, F., Clarke, J., Mansfield, W., et al. (2014). Resetting transcription factor control circuitry toward ground-state pluripotency in human. Cell *158*, 1254–1269.

Theunissen, T.W., Friedli, M., He, Y., Planet, E., O'Neil, R.C., Markoulaki, S., Pontis, J., Wang, H., Iouranova, A., Imbeault, M.,

et al. (2016). Molecular criteria for defining the naive human pluripotent state. Cell Stem Cell *19*, 502–515.

Theunissen, T.W., Powell, B.E., Wang, H., Mitalipova, M., Faddah, D.A., Reddy, J., Fan, Z.P., Maetzel, D., Ganz, K., Shi, L., et al. (2014). Systematic identification of culture conditions for induction and maintenance of naive human pluripotency. Cell Stem Cell *15*, 471–487.

Thomson, J.A., Itskovitz-Eldor, J., Shapiro, S.S., Waknitz, M.A., Swiergiel, J.J., Marshall, V.S., and Jones, J.M. (1998). Embryonic stem cell lines derived from human blastocysts. Science *282*, 1145–1147.

Trusler, O., Huang, Z., Goodwin, J., and Laslett, A.L. (2018). Cell surface markers for the identification and study of human naive pluripotent stem cells. Stem Cell Res. *26*, 36–43.

Ware, C.B., Nelson, A.M., Mecham, B., Hesson, J., Zhou, W., Jonlin, E.C., Jimenez-Caliani, A.J., Deng, X., Cavanaugh, C., Cook, S., et al. (2014). Derivation of naive human embryonic stem cells. Proc. Natl. Acad. Sci. U S A *111*, 4484–4489.

Yang, Y., Liu, B., Xu, J., Wang, J., Wu, J., Shi, C., Xu, Y., Dong, J., Wang, C., Lai, W., et al. (2017). Derivation of pluripotent stem cells with in vivo embryonic and extraembryonic potency. Cell *169*, 243–257.e25.

Stem Cell Reports, Volume *12*

Supplemental Information

The Cell-Surface Marker Sushi Containing Domain 2 Facilitates Establishment of Human Naive Pluripotent Stem Cells

Nicholas Bredenkamp, Giuliano Giuseppe Stirparo, Jennifer Nichols, Austin Smith, and Ge Guo

INVENTORY OF SUPPLEMENTAL INFORMATION

Supplemental methods

Cell culture procedures, reverse transcription and RT-qPCR, immunofluorescence staining, and flow cytometry are described.

Table S1 RT-qPCR assays

Supplemental figure legends

Figure S1 Cell surface marker expression by RNA-seq analysis, Relates to Figure 1

Figure S2 Cell surface marker expression in human embryos and naïve hPSC, Relates to Figure 1

Figure S3 Capacitation of human naïve PSC, Relates to Figure 1

Figure S4 Application of SUSD2 for identification and purification of human naïve PSC during resetting and reprogramming, Relates to Figure 2 and Figure 4

Supplemental references

SUPPLEMENTAL METHODS

Human naïve PSC culture

Unless otherwise indicated, human naïve PSC were propagated in PXGL medium on irradiated MEF feeders. ROCK inhibitor (Y-27632) was added during replating. Geltrex (10 μg per cm^2 surface area) was added to media during plating for feeder-free expansion.

PXGL medium: N2B27 (in house) supplemented with 1 μM PD0325901 (P), 2μM XAV939 (X), 2 μM Gö6983 (G) and 10 ng/ml human LIF (L).

t2iLGö medium: N2B27 supplemented with 1 μM PD0325901, 1 μM CHIR99021 (2i), 2 μM Gö6983 (Gö) and 10 ng/ml human LIF (L).

N2B27 medium (1L): 487 ml DMEM/F12 (Sigma, D6421), 487 ml Neurobasal (Thermo Fisher, 21103049), 10 ml B27 (Thermo Fisher, 17504044), 5 ml N2 (made in house), 10 ml L-Glutamine (200mM, Thermo Fisher, 25030024), 1ml 0.1M β-mercaptoethanol (Sigma, M3148)

N2 (made in house): DMEM/F12 basal medium supplemented with 0.4 mg/ml Insulin (Sigma, 19278), 10 mg/ml Apo-transferrin (eBioscience, ABC2553), 3 μM Sodium selenite (Sigma, S5261), 1.6 mg/ml Putrescine (Sigma, P5780) and 2 μg/ml Progesterone (Sigma, P8783).

Chemical Resetting

Conventional human PSC were seeded at $1\times10^4/cm^2$ onto irradiated MEF feeders in E8 medium. Two days later (day 0) medium was changed to PDL/HDACi (N2B27 with 1μM PD0325901, 10ng/ml human LIF and 1mM valproic acid sodium salt (VPA, Sigma, P4543) (HDACi)). Following 3 days in PDL/HDACi, medium was changed to

PXGL and refreshed daily for a further 10-11 days before passaging to establish naïve PSC cultures in PXGL medium on irradiated MEF feeders.

Somatic cell reprogramming

Human dermal fibroblast (HDF) cells were seeded onto gelatin coated plates in fibroblast culture medium at a density of 1×10^4 cells per cm^2. The next day Sendai virus vectors (Fusaki et al., 2009) were applied for 24 hours and the culture was refreshed with fibroblast culture medium. Following 3 or 4 more days of culture in fibroblast culture medium, cells were passaged onto irradiated mouse MEF feeders and cultured in Essential 6 (E6) supplemented with 10ng/ml FGF2 (prepared in-house) for 4 days. Cells were then switched into naïve media (PXGL+Y) to capture naïve iPSCs. Episomal reprogramming was performed with similar protocol except that reprogramming factors (*pCXLE-OCT4-shRNA(p53*), *pCXLE--SOX2-KLF4* and *pCXLE-L-MYC-LIN28* (Okita et al., 2011) were transfected into HDFs by electroporation.

Reverse transcription and RT-qPCR

Total RNA was extracted using a RNeasy Kit (Qiagen) and cDNA synthesized with SuperScript III reverse transcriptase (Thermo Fisher Scientific, 18080085) and oligo(dT) adapter primers. TaqMan assays and Universal Probe Library (UPL) probes (Roche Molecular Systems) were used to perform gene quantification.

Immunostaining

Cells were fixed with 4% PFA for 15 min at room temperature and blocked/permeabilised with 0.1% Triton X-100 and 5% donkey serum in PBS for 30

min. Incubation with primary antibodies KLF17 (Atlas Antibodies HPA024629), NANOG (R&D Systems AF1997), TFCP2L1 (R&D Systems AF5726), SUSD2 clone W5C5 (BioLegend 327401), GATA4 (Santa Cruz sc-1237), MECP2 (Cell Signalling Technology 3456T) was overnight at 4°C and secondary antibodies were added for 1 h at room temperature. For live cell staining of human cells incubation with conjugated SUSD2 clone W5C5 (SUSD2-PE, BioLegend 327406; SUSD2-FITC, Miltenyi Biotech 130-106-401), CD7-PE clone 6B7 (BioLegend 343105), CD75-FITC clone LN1 (BD Biosciences 555654), CD77-FITC clone 5B5 (BioLegend 357103), CD130-PE clone 2E1B02 (BioLegend 362003) in culture media was for 30 min before washing and imaging.

Flow cytometry

Flow cytometry analysis was carried out on a Fortessa instrument (BD Biosciences). Cell sorting was performed using a MoFlo high-speed instrument (Beckman Coulter). The following antibodies were used for flow cytometry: SUSD2 clone W5C5 (SUSD2-PE, BioLegend 327406; SUSD2-APC, BioLegend 327408) CD24-FITC (BioLegend 311104) or CD24-APC or CD24-PECy7 (eBioscience 17-0247-42 or 25-0247-41), SSEA4-APC (BioLegend 330418), CD7-APC (BioLegend 343108), CD75-eFluor 660 (eBioscience 50-0759-42), CD77-Alexa 647 (BD Biosciences 563632), CD130-PE (BioLegend 362003).

Table S1. RT-qPCR assays

GENE	ASSAY METHOD	DETAILS
ACTB	TaqMan	Assay ID: Hs01060665_g1
NANOG	TaqMan	Assay ID: Hs02387400_g1
OCT4	TaqMan	Assay ID: Hs01654807_s1
KLF4	TaqMan	Assay ID: Hs00358836_m1
KLF17	TaqMan	Assay ID: Hs00703004_s1
TFCP2L1	TaqMan	Assay ID: Hs00232708_m1
DPPA3	TaqMan	Assay ID: Hs01931905_g1
DPPA5	TaqMan	Assay ID: Hs00988349_g1
SUSD2	UPL	Forward primer: agagctggatggacctgaaa<br>Reverse primer: atgccagcatgatggagac<br>UPL probe number: 83

SUPPLEMENTAL FIGURE LEGENDS

Figure S1 Cell surface marker expression by RNA-seq analysis, Relates to Figure 1

(A) Published human naïve cell surface marker (Collier et al., 2016; O'Brien et al., 2016) transcript levels in human embryos at different stages and lineages shown, and in various naïve (pale pink bars) and conventional (green bars) hPSC extracted from an integrated single cell RNA-sequence dataset (Stirparo et al., 2018). cMOR, compacted morula; eICM, early inner cells mass; TE, trophectoderm; Epi, epiblast; PrE, primitive endoderm. (B) Epiblast to primitive endoderm average FPKM ratio for published cell surface markers and SUSD2.

Figure S2 Cell surface marker expression in human embryos and naïve hPSC, Relates to Figure 1

(A) Immunofluorescence staining for SUSD2, KLF17 and GATA4 in three E7 human blastocysts; KLF17 marks the ICM. Scale bar, 50 µm. (B) RT-qPCR analysis of general

and naïve pluripotency markers in HNES1 cells in t2iLGö or PXGL medium. (C) Top panel, flow cytometry analysis showing cell surface marker expression in conventional (SHEF6, S6), naïve chemically reset hPSC (cR-S6) and naïve embryo derived hPSC (HNES1). Bottom panel, images show bright field and immunofluorescence staining using conjugated antibodies as shown, on live naïve chemically reset hPSC (cR-H9) and naïve embryo derived hPSC (HNES1). Exposure time: CD7-PE, CD75-FITC, CD77-FITC, CD130-PE, 15s; SUSD2-FITC, 12s; SUSD2-PE, 3s. Scale bar, 100μm.

Figure S3 Capacitation of human naïve PSC, Relates to Figure 1

(A) Bright field images show colony morphology changes during capacitation of cR-S6 and HNES1 cells in N2B27-XAV medium. Scale bars, 50μm. (B) Alkaline phosphatase (AP) staining of cR-S6 and HNES1 cells following 10 days capacitation and replating and culture in PXGL for 7 days. Images show AP staining of a single well of a 12-well tissue culture plate seeded with 12500 capacitated or parental naive cells.

Figure S4 Application of SUSD2 for identification and purification of human naïve PSC during resetting and reprogramming, Relates to Figure 2 and Figure 4

(A) Flow cytometry analysis showing SUSD2 and CD24 expression of cell populations five days after sorting. $SUSD2^+$, $SUSD2^+CD24^-$; $SUSD2^-$, $SUSD2^-CD24^+$. Bright field and GFP images of cell populations at passage one and five after sorting. Scale bar, 50μm. (B) RT-qPCR analysis of general and naïve pluripotency markers in sorted $SUSD2^+CD24^-$ and $SUSD2^-CD24^+$ cells at P1, P3 and P10 after resetting. Error bars indicate s.d. of three biological replicates. (C) Flow cytometry analysis showing

SUSD2 and CD24 expression in cR-H9-EOS cells at passage 5 and 10. Left column shows unsorted reset cultures, bottom row shows cultures that were sorted for SUSD2^{+}CD24^{-} at day 14 of resetting. (D) Images show bright field and immunofluorescence staining for SUSD2 on live cultures during episomal reprograming. Scale bar, 50µm. (E) Flow cytometry analysis showing SUSD2, EPCAM and CD24 expression during episomal reprograming. (F) Immunofluorescence staining for SUSD2 and KLF17 during episomal reprograming.

REFERENCES

Fusaki, N., Ban, H., Nishiyama, A., Saeki, K., and Hasegawa, M. (2009). Efficient induction of transgene-free human pluripotent stem cells using a vector based on Sendai virus, an RNA virus that does not integrate into the host genome. Proc Jpn Acad Ser B Phys Biol Sci *85*, 348-362.

Okita, K., Matsumura, Y., Sato, Y., Okada, A., Morizane, A., Okamoto, S., Hong, H., Nakagawa, M., Tanabe, K., Tezuka, K*., et al.* (2011). A more efficient method to generate integration-free human iPS cells. Nat Methods *8*, 409-412.

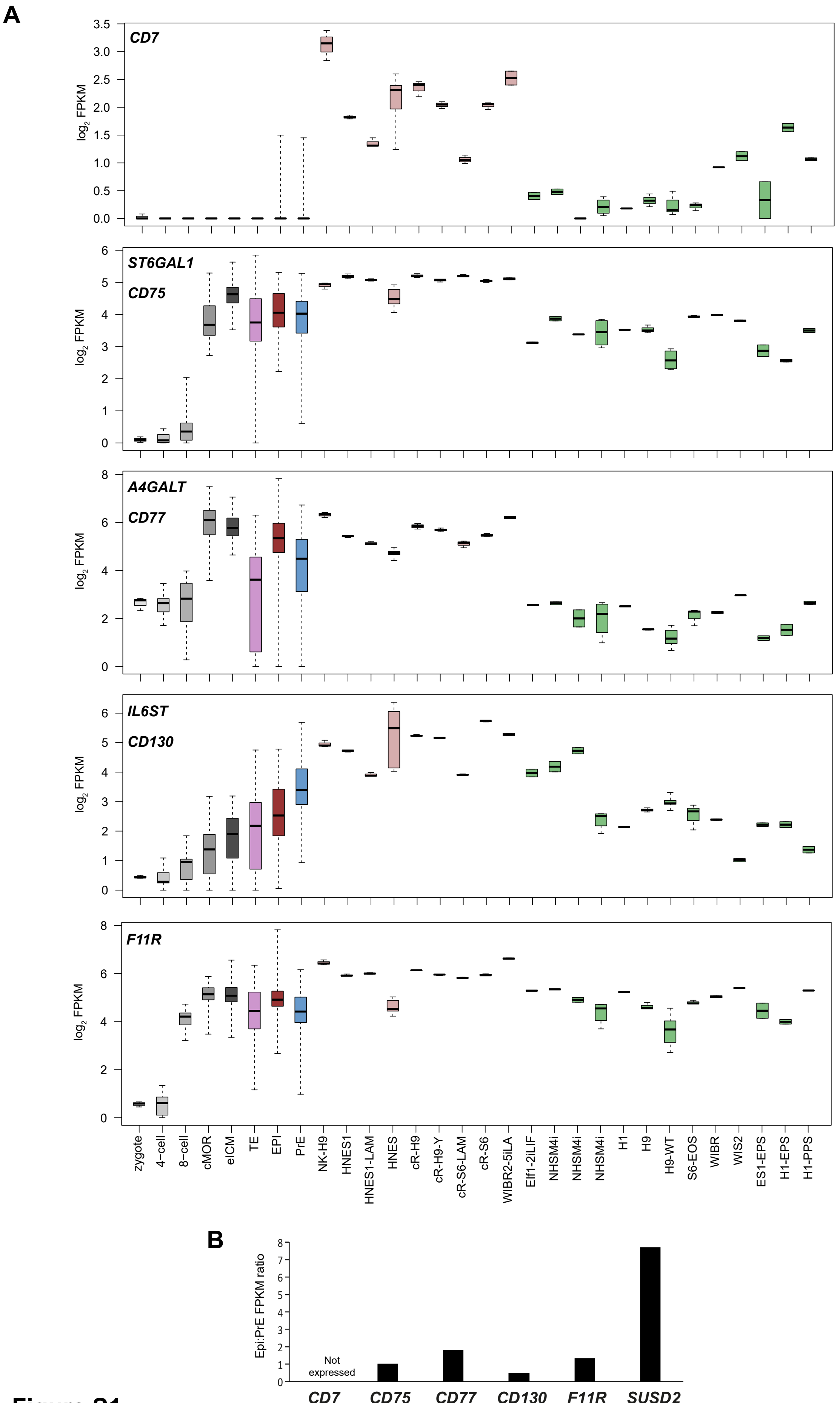
A
CD7
ST6GAL1
CD75
A4GALT
CD77
IL6ST
CD130
F11R
log2 FPKM
zygote
4-cell
8-cell
cMOR
eICM
TE
EPI
PrE
NK-H9
HNES1
HNES1-LAM
HNES
cR-H9
cR-H9-Y
cR-S6-LAM
cR-S6
WIBR2-5iLA
EIf1-2iLIF
NHSM4i
NHSM4i
NHSM4i
H1
H9
H9-WT
S6-EOS
WIBR
WIS2
ES1-EPS
H1-EPS
H1-PPS
B
Epi:PrE FPKM ratio
Not expressed
CD7
CD75
CD77
CD130
F11R
SUSD2

Figure S1

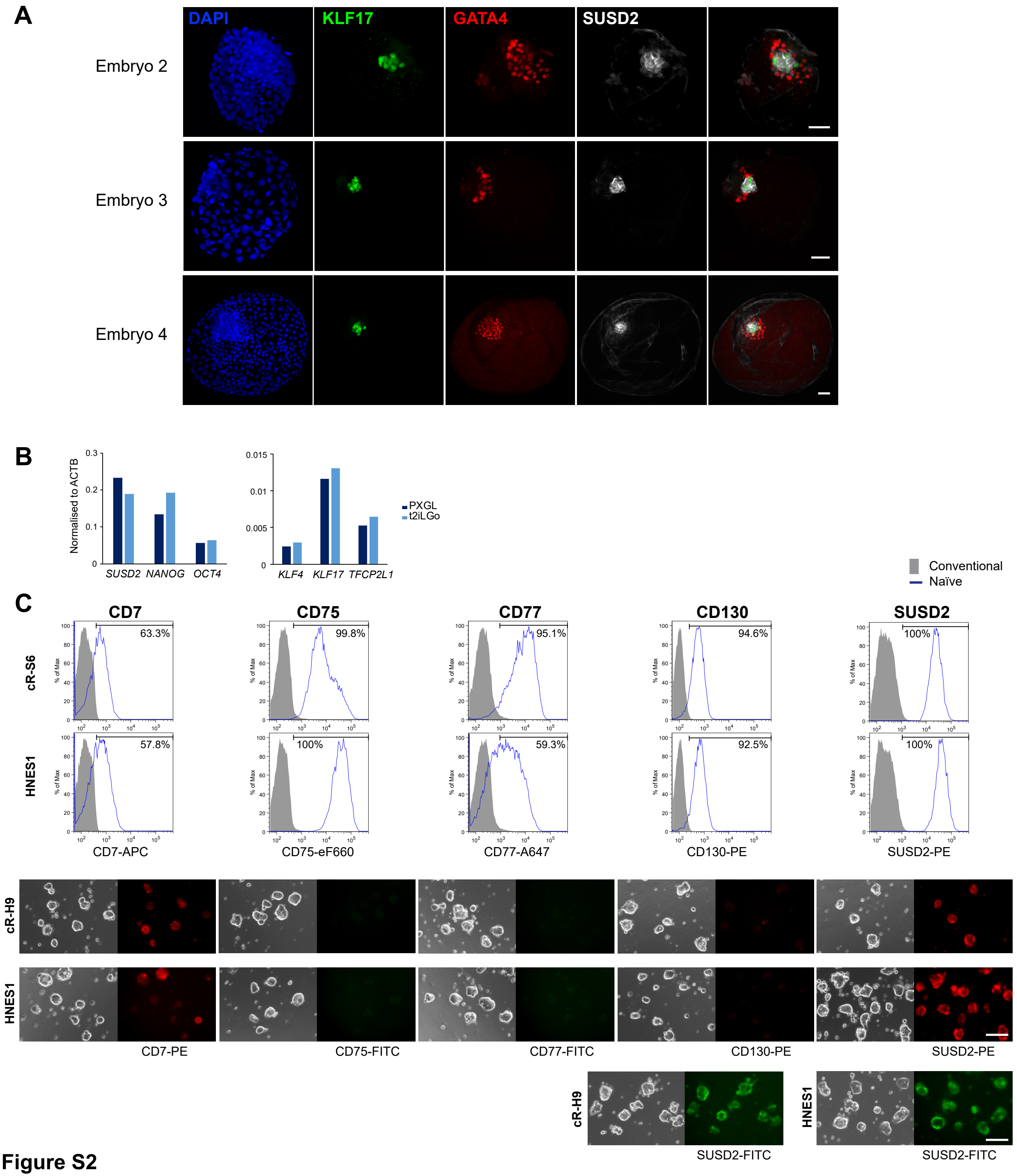
A
DAPI
KLF17
GATA4
SUSD2
Embryo 2
Embryo 3
Embryo 4
B
Normalised to ACTB
SUSD2
NANOG
OCT4
KLF4
KLF17
TFCP2L1
PXGL
t2iLGo
C
Conventional
Naïve
CD7
CD75
CD77
CD130
SUSD2
cR-S6
HNES1
63.3%
99.8%
95.1%
94.6%
100%
57.8%
100%
59.3%
92.5%
100%
% of Max
CD7-APC
CD75-eF660
CD77-A647
CD130-PE
SUSD2-PE
cR-H9
HNES1
CD7-PE
CD75-FITC
CD77-FITC
CD130-PE
SUSD2-PE
cR-H9
HNES1
SUSD2-FITC
SUSD2-FITC

Figure S2

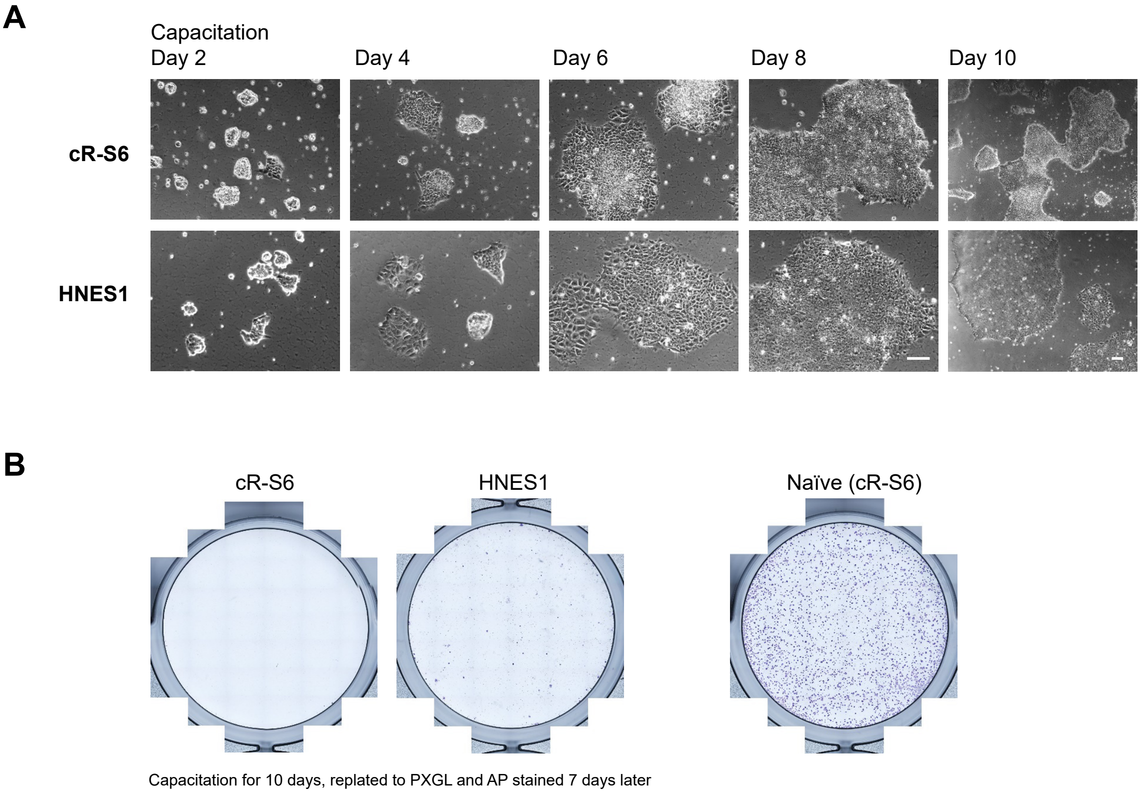
A
Capacitation
Day 2
Day 4
Day 6
Day 8
Day 10
cR-S6
HNES1
B
cR-S6
HNES1
Naïve (cR-S6)
Capacitation for 10 days, replated to PXGL and AP stained 7 days later

Figure S3

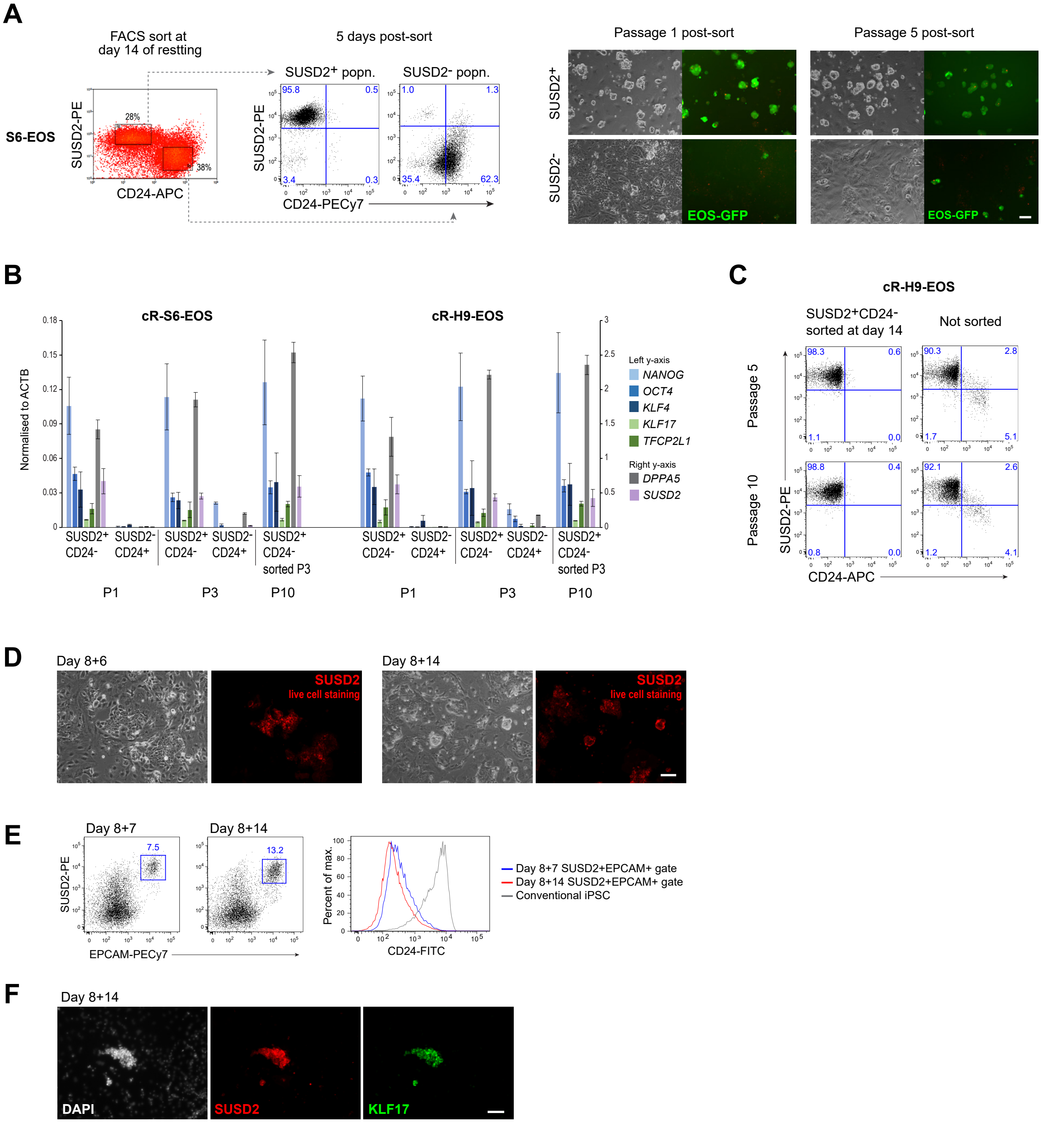
A
FACS sort at day 14 of restting
5 days post-sort
SUSD2+ popn.
SUSD2- popn.
S6-EOS
SUSD2-PE
CD24-APC
28%
38%
CD24-PECy7
95.8
0.5
3.4
0.3
1.0
1.3
35.4
62.3
Passage 1 post-sort
Passage 5 post-sort
SUSD2+
SUSD2-
EOS-GFP
B
cR-S6-EOS
cR-H9-EOS
Normalised to ACTB
Left y-axis
NANOG
OCT4
KLF4
KLF17
TFCP2L1
Right y-axis
DPPA5
SUSD2
SUSD2+ CD24-
SUSD2- CD24+
SUSD2+ CD24- sorted P3
P1
P3
P10
C
cR-H9-EOS
SUSD2+CD24- sorted at day 14
Not sorted
Passage 5
Passage 10
SUSD2-PE
CD24-APC
98.3
0.6
1.1
0.0
90.3
2.8
1.7
5.1
98.8
0.4
0.8
0.0
92.1
2.6
1.2
4.1
D
Day 8+6
Day 8+14
SUSD2
live cell staining
E
Day 8+7
Day 8+14
7.5
13.2
SUSD2-PE
EPCAM-PECy7
Percent of max.
CD24-FITC
Day 8+7 SUSD2+EPCAM+ gate
Day 8+14 SUSD2+EPCAM+ gate
Conventional iPSC
F
Day 8+14
DAPI
SUSD2
KLF17

Figure S4
